# Cryptic Species in Tropic Sands - Interactive 3D Anatomy, Molecular Phylogeny and Evolution of Meiofaunal Pseudunelidae (Gastropoda, Acochlidia)

**DOI:** 10.1371/journal.pone.0023313

**Published:** 2011-08-31

**Authors:** Timea P. Neusser, Katharina M. Jörger, Michael Schrödl

**Affiliations:** 1 Bavarian State Collection of Zoology, München, Germany; 2 Department Biology I of the Ludwig-Maximilians-Universität München, Planegg-Martinsried, Germany; Public Library of Science, United Kingdom

## Abstract

**Background:**

Towards realistic estimations of the diversity of marine animals, tiny meiofaunal species usually are underrepresented. Since the biological species concept is hardly applicable on exotic and elusive animals, it is even more important to apply a morphospecies concept on the best level of information possible, using accurate and efficient methodology such as 3D modelling from histological sections. Molecular approaches such as sequence analyses may reveal further, cryptic species. This is the first case study on meiofaunal gastropods to test diversity estimations from traditional taxonomy against results from modern microanatomical methodology and molecular systematics.

**Results:**

The examined meiofaunal *Pseudunela* specimens from several Indo-Pacific islands cannot be distinguished by external features. Their 3D microanatomy shows differences in the organ systems and allows for taxonomic separation in some cases. Additional molecular analyses based on partial mitochondrial cytochrome *c* oxidase subunit I (COI) and 16S rRNA markers revealed considerable genetic structure that is largely congruent with anatomical or geographical patterns. Two new species (*Pseudunela viatoris* and *P. marteli* spp. nov.) are formally described integrating morphological and genetic analyses. Phylogenetic analysis using partial 16S rRNA, COI and the nuclear 18S rRNA markers shows a clade of Pseudunelidae species as the sister group to limnic Acochlidiidae. Within *Pseudunela*, two subtypes of complex excretory systems occur. A complex kidney already evolved in the ancestor of Hedylopsacea. Several habitat shifts occurred during hedylopsacean evolution.

**Conclusions:**

Cryptic species occur in tropical meiofaunal *Pseudunela* gastropods, and likely in other meiofaunal groups with poor dispersal abilities, boosting current diversity estimations. Only a combined 3D microanatomical and molecular approach revealed actual species diversity within *Pseudunela* reliably. Such integrative methods are recommended for all taxonomic approaches and biodiversity surveys on soft-bodied and small-sized invertebrates. With increasing taxon sampling and details studied, the evolution of acochlidian panpulmonates is even more complex than expected.

## Introduction

The study of cryptic species, i.e. two or more distinct species classified as a single species due to the lack of morphological differences, augmented during the last 20 years [1]. There is a consensus about the importance of our knowledge of cryptic diversity for, amongst others, animal diversity estimations, biological control, natural resource protection and conservation (e.g. [1], [2]). However, the distribution of cryptic species among metazoan taxa and biogeographical regions is discussed controversially. Whereas Bickford et al. [1] proposed a non-random distribution across taxa and biomes, Pfenninger & Schwenk [3] suggested an almost even distribution among the major metazoan taxa and biogeographical regions. Trontelj & Fiser [2] emphasised that regularities of the cryptic diversity probably will be discovered only by means of genus- or species-level studies.

One area with an unexpectedly high level of cryptic speciation is the Antarctic Ocean. Molecular studies revealed flocks of cryptic rather than single widespread and variable species throughout benthic invertebrate groups examined, e.g. in crinoids, pycnogonids, crustaceans and molluscs [4], [5], [6], [7]. Many, but not all of those organisms from high geographic latitudes are brooders or direct developers with low dispersal abilities, such as the nudibranch gastropod *Doris kerguelenensis* (Bergh, 1884) which ultimately was shown to have undergone an explosive cryptic radiation in the Southern Ocean [6]. According to Thorson's rule, direct developers in benthic organisms such as most molluscs are considered as scarce in subtropical or tropical waters [8]. Exceptions are members of taxa living in the mesopsammon which generally are assumed to be direct developers [9] or, as in case of acochlidian panpulmonate gastropods, may have planktonic larvae which remain in the interstitial spaces [10]. Thus, it can be assumed that their dispersal ability in the larval stage is very low. Also, meiofaunal acochlidian gastropods appear to occur in coastal sands only, i.e. postlarval stages have virtually no potential for active migration or forming continuous populations across deeper waters. Given this level of supposed immobility and habitat restrictions as opposed to the vast coasts of the world's oceans and innumerable, highly isolated archipelagos and off-shore reefs we should expect that there are plenty of narrow ranged rather than a few wide-ranged acochlidian species. However, based on morphology, only 28 valid species, 20 of them mesopsammic, were described globally. Several of these species such as *Microhedyle remanei* (Marcus, 1953) were considered to be widespread throughout Western Atlantic warm water sands, i.e. in Brazil, Colombia and Bermuda [11], [12], [13], [14], and *Pseudunela cornuta* (Challis, 1970) was recorded to occur on the Solomon Islands (Melanesia) and near Hong Kong (South China Sea) [15], [16]. Recently, both species were re-described in considerable anatomical and histological detail [14], [17]. However, until now, applying morphospecies concepts on tiny meiofaunal gastropods has never been tested by molecular analyses.

During several expeditions to different Indo-Pacific archipelagos and islands, specimens of the genus *Pseudunela* have been collected and preserved for comparative structural and molecular investigation. Externally, they show variation regarding the colour of the digestive gland shining through the epidermis and the external identification of the eyes, but both features do not allow an unambiguous discrimination from the well-described *P. cornuta* from the Solomon Islands. Within the Hedylopsacea the marine and brackish genus *Pseudunela* possesses a key position as sister group to the limnic Acochlidiidae [18]. For a better understanding of the invasion of freshwater systems and the evolution of involved organ systems in Acochlidia, it was thus indispensable to assess the organ and species diversity within *Pseudunela*, as well as their phylogeny and directions of evolution. *Pseudunela cornuta* from the Solomon Islands was first described by Challis [15]. Recently, these original data were complemented and corrected by Neusser et al. [17] including an interactive 3D-reconstruction. Hughes [16] reported of a second record of *P. cornuta* from Hong Kong. However, her species description is very brief and vague, so that a recollection at the same locality and a detailed re-description of this species is essential before including it in our comparative study of *Pseudunela*. The same situation applies to the description of *Pseudunela eirene* Wawra, 1988 [19] which needs a revision as well.

The present study gives an extensive anatomical description of all *Pseudunela* specimens available to us, including interactive 3D-reconstructions of *Pseudunela viatoris* sp. nov. from Fiji. Another new species involved is described in the same detail in the present study and is briefly compared with *P. viatoris* sp. nov.. The genetic diversity within *Pseudunela* is assessed using partial mitochondrial cytochrome *c* oxidase subunit I (COI) gene, which was proposed as standard DNA barcoding marker [20], [21], [22], and partial 16S rRNA gene sequences. The origin and the phylogenetic relationships of *Pseudunela* species are reconstructed by additionally using the nuclear 18S rRNA marker. The largely cryptic radiation of the different *Pseudunela* species is discussed. A possible scenario on the evolution of the excretory system in Acochlidia is given.

## Methods

### Sampling and semithin sectioning

Specimens of different *Pseudunela* species were collected during expeditions to various Indo-Pacific Islands, namely Fiji, Indonesia, Solomon Islands and Vanuatu. They were extracted from sand samples according to Schrödl [23] and subsequently relaxed by a solution of isotonic MgCl_2_. Some specimens were preserved in 4% glutardialdehyde in 0.2 M sodium cacodylate buffer (0.1 M NaCl and 0.35 M sucrose, pH 7.2), followed by post-fixation in buffered 1% OsO_4_ for 1.5 h in the dark. The specimens were decalcified in 1% ascorbic acid overnight and dehydrated in an acetone series (30, 50, 70, 90, 100%). For semithin sectioning specimens were embedded in Spurr's low viscosity resin [24]. Several series of ribboned serial semithin sections of 1.5 µm thickness were prepared using a diamond knife (Histo Jumbo, Diatome, Biel, Switzerland) and contact cement on the lower cutting edge to form ribbons [25]. Sections finally were stained with methylene-azure II [26] and were deposited at the Mollusca Department, Bavarian State Collection of Zoology (ZSM), Munich, Germany. A list of the material examined including the museum numbers is shown in Table 1.

**Table 1:** Material examined in the present study.

Species	Locality	MuseumN°	Pre- paration type	Accession number of DNA voucher (ZSM)	GenBank Accession N°		
					COI	16S	18S
*Pseudunela viatoris* sp. nov.	Fiji, Viti Levu, Laucala Bay, Nukumbutho Island	20080492	sections				
		20080493	sections				
		20062048	SEM				
		20080020	mol	AB34404247	JF819766	JF819741	JF819751
		20080021	mol	AB34404265	JF819767	JF819742	-
		20080057	mol	AB34404281	JF819768	JF819743	-
*Pseudunela viatoris* sp. nov.	Indonesia, bay of Gili Lawa Laut Island	20090422	sections				
		20090423	sections				
		20071120	SEM				
		20071120	mol	AB34404285	JF819769	JF819744	JF819752
		20070953	mol	AB34404276	JF819770	JF819745	-
*Pseudunela marteli* sp. nov.	Solomon Islands, Guadalcanal, Honiara, beach of “Art Gallery”	20071851	sections				
		20071864	sections				
		20071865	sections				
		20071826	SEM				
		20080022	mol	AB34404252	JF819771	JF819746	JF819753
		20080023	mol	AB34404298	JF819772	-	-
		20080024	mol	AB34404218	JF819773	JF819747	-
*Pseudunela marteli* sp. nov.	Vanuatu, Oyster Island	20071061	sections				
		20090416	sections				
		20080105	SEM				
		20080393	GenBank	AB35081809	HQ168456	HQ168418	HQ168431
*Pseudunela cornuta*	Solomon Islands, Guadalcanal, Komimbo Bay	20071809	mol	AB34404215	JF819774	JF819748	JF819754
*Pseudunela espiritusanta*	Vanuatu, Espiritu Santo	20080117	mol	AB34404289	JF819775	JF819749	JF819755
		20071118	mol	AB34404210	JF819776	JF819750	-
*Hedylopsis ballantinei*	Egypt, Dahab, Red Sea	20090244	GenBank	AB34858170	HQ168454	HQ168416	HQ168429
*Strubellia paradoxa*	Indonesia, Ambon, Maluku Utara	193944 (Natural History Museum, Berlin)	GenBank	AB34858174	HQ168457	HQ168419	HQ168432
*Acochlidium fijiense*	Fiji, Viti Levu, Lami River	20080063	GenBank	AB34404244	HQ168458	HQ168420	HQ168433
*Microhedyle glandulifera*	Croatia, Istria, Kap Kamenjak	20081019	GenBank	AB35081799	HQ168461	HQ168424	HQ168437
Aitengidae sp.	Japan, Okinawa, Miyako Island	-	GenBank	-	HQ168453	HQ168415	HQ168428

Museums numbers refer to the Bavarian State Collection of Zoology, Germany (**ZSM**), if not indicated otherwise; **GenBank**, molecular data retrieved from GenBank; **mol**, molecular data generated within this study; **sections**, semithin serial sections for histology; **SEM**, scanning electron microscopy.

### 3D reconstruction

Digital photographs of every slice were taken with a CCD microscope camera (Spot Insight, Diagnostic Instruments, Sterling Heights, USA) mounted on a DMB-RBE microscope (Leica Microsystems, Wetzlar, Germany). Images were converted to 8bit greyscale format, contrast enhanced and unsharp masked with standard image editing software. A detailed computer-based 3D-reconstruction of all major organ systems was conducted with the software AMIRA 5.2 (Visage Imaging GmbH, Berlin, Germany) following basically the procedure explained by Ruthensteiner [25]. The presented 3D-reconstruction is based on series N° ZSM 20080492.

### Interactive 3D-model

The interactive 3D-model for the supporting information was prepared according to Ruthensteiner & Heß [27], but using different software, i.e. the 3D tools of Deep Exploration 5.5 (Right Hemisphere EMEA, Germany) and Adobe Acrobat 9.0 Professional Extended (Adobe Systems GmbH, Germany). The reconstructed surfaces were saved as *.obj format in Amira and one by one opened in Deep Exploration. The display settings were adjusted (solid, no grid, CAD optimized illumination, smoothing 180°) and each surface was reduced to 10–30%. The surfaces were saved as *.u3d format. Finally, a complex *.u3d model including all surfaces was generated. For that purpose each surface was given a name and colour and the model was set up using the function ‘merge file’. The surfaces were arranged according to organ systems using the function ‘create group’. The *.u3d model was imported in a pdf in Adobe Acrobat 9.0 Professional Extended and different views of the organ systems were prefabricated to standard views allowing the reader to get rapidly a general idea of the model. The 3D-model is accessible by clicking onto the figure in the supporting information figure S1 (Adobe Reader Version 7 or higher required).

### Analysis by scanning electron microscopy (SEM)

Specimens preserved in 75% and 96% EtOH were used for the examination of the radulae by SEM. They were macerated in 10% KOH overnight to separate the radula from the surrounding tissue. Remaining tissue was manually removed with fine dissection pins. The radulae were mounted on specimen stubs, sputter coated with gold for 135 sec. (SEM-Coating-System, Polaron) and analysed using a LEO 1430 VP (Leo Elektronenmikroskopie GmbH, Oberkochen, Germany) at 15 kV.

### DNA extraction, polymerase chain reaction and sequencing

DNA was extracted from entire specimens using QIAGEN DNeasy Tissue Kit according to the manufacture's instructions. Three different gene regions were amplified: approximately 650 bp of the mitochondrial cytochrome *c* oxidase subunit I (COI) gene; partial mitochondrial 16S rRNA gene sequence (around 420 bp) and approximately 1800 bp of the nuclear 18S rRNA gene (for PCR protocols and primers used see Table 2). Successful PCR products were cleaned up using ExoSapIT (USB, Affymetrix, Inc.). Cycle sequencing and the sequencing reaction was performed by the sequencing service of the Department of Biology Genomic Service Unit (GSU) of the Ludwig-Maximilians-University Munich using Big Dye 3.1 kit and an ABI 3730 capillary sequencer. All fragments were sequenced in both directions using the PCR primers as specified in Table 2.

**Table 2:** Primer sequences and PCR protocols used for each of the amplified gene regions.

Gene region	Primer	Sequence 5′ - 3′	Reference	PCR program
**18S**	18A1	CCT ACT TCT GGT TGA TCC TGC CAG T	[70]	98°C 30 sec (98°C 5 sec, 48–65°C 5 sec, 72°C 20–25 sec)×28–40, 72°C 60 sec(Phire polymerase, New England Biolabs)
	700R	CGC GGC TGC TGG CAC CAG AC	[71]	
	470F	CAG CAG GCA CGC AAA TTA CCC	[71]	
	1500R	CAT CTA GGG CAT CAC AGA CC	[71]	
	1155F	CTG AAA CTT AAA GGA ATT GAC GG	[71]	
	1800	TAA TGA TCC TTC CGC AGG TT	[70]	
**16S**	16S-H	CGC CTG TTT ATC AAA AAC AT	[72]	98°C 30 sec (98°C 5 sec, 48–55°C 5 sec, 72°C 25 sec)×35–40, 72°C 60 sec(Phire polymerase, New England Biolabs)
	16S-R	CCG GTC TGA ACT CAG ATC ACG T	[72]	
	16Sf-50	GGC CGC AGT ACC TTG ACT GT	present study	
	16Sr-380	TCC ACC ATC GAG GTC ACA AG	present study	
**COI**	LCO1490	GGT CAA CAA ATC ATA AAG ATA TTG G	[73]	94°C 3 min (94°C 60 sec, 48–52°C 60 sec, 72°C 90 sec)×35–40, 72°C 3 min(Taq polymerase, Sigma)
	HCO2198	TAA ACT TCA GGG TGA CCA AAA AAT CA	[73]	

For 16S rRNA gene and COI one to three individual(s) of each *Pseudunela* species were sequenced and analysed, for 18S rRNA gene and outgroup species only one specimen was analysed. Outgroup sequences were retrieved from GenBank (see Table 1) and selected based on the latest phylogenetic hypotheses of the Acochlidia [18], [28]. All sequences generated within this study are deposited to GenBank and DNA aliquots are stored at DNAbank at the ZSM (http://www.dnabank-network.org) (see Table 1 for accession numbers).

### Sequence alignment and phylogenetic analyses

All sequences generated were checked for contaminations with BLAST searches [29], implemented in the GenBank database. Sequences were edited using BioEdit 7.0.9 and Sequencher 4.8 (Gene Codes Corporation). The alignment was performed with MAFFT v6 [30] using the default settings. The alignment of the protein-coding COI data was corrected manually according to amino acids. Poorly aligned positions and divergent regions in the 18S rRNA gene and 16S rRNA gene alignment were excluded using the standard options for a less stringent selection in Gblocks [31].

The combined data set comprised of the 18S, 16S and COI was subject to phylogenetic analyses using maximum likelihood in RAxML 7.0.4 [32]. Data were analysed in four partitions (18S; 16S; COI 1^st^ and 2^nd^ codon position and 3^rd^ separately) under the G+Γ+I model selected with jModeltest [33]. The microhedylacean *Microhedyle glandulifera* was defined as outgroup, following recent phylogenetic approaches based on morphology [18] and molecular data [28]. The program parameters were adapted to the alignment as described in the manual (“hard and slow way” – with ten parsimony starting trees and six different rate categories). Additionally 200 multiple interferences were executed on the alignment and 1000 bootstrap replicates were generated.

For species delineation based on our molecular dataset, we additionally used Species Identifier (obtained from TaxonDNA [34]) to group sequences into clusters based on pairwise distances of both mitochondrial markers (testing thresholds from 1–10%) and to evaluate intra- and interspecific variation. Haplotype networks of *Pseudunela* based on the partial mitochondrial COI sequences were inferred using statistical parsimony as implemented in TCS 1.21 [35] under the default settings (95% confidence criterion) for both mitochondrial markers. Using a maximum likelihood approach, the general mixed Yule-coalescent (GMYC) model is able to discriminate between population and speciation patterns based on a phylogenetic tree (for detailed description of the methodology see [36], [37]). We performed GMYC using the R package SPLITS (http://r-forge.r-project.org/projects/splits/). The input tree was generated with RAxML 7.0.4 [32] as described above, based on the concatenated mitochondrial dataset (COI+16S). Our RAxML tree was converted into an ultrametric tree using the package ‘ape’ in R (chronopl function [38]) and an analysis allowing multiple thresholds [36] was performed.

### Nomenclatural acts

The electronic version of this document does not represent a published work according to the International Code of Zoological Nomenclature (ICZN), and hence the nomenclatural acts contained in the electronic version are not available under that Code from the electronic edition. Therefore, a separate edition of this document was produced by a method that assures numerous identical and durable copies, and those copies were simultaneously obtainable (from the publication date noted on the first page of this article) for the purpose of providing a public and permanent scientific record, in accordance with Article 8.1 of the Code. The separate print-only edition is available on request from PLoS by sending a request to PLoS ONE, Public Library of Science, 1160 Battery Street, Suite 100, San Francisco, CA 94111, USA along with a check for $10 (to cover printing and postage) payable to “Public Library of Science”.

In addition, this published work and the nomenclatural acts it contains have been registered in ZooBank, the proposed online registration system for the ICZN. The ZooBank LSIDs (Life Science Identifiers) can be resolved and the associated information viewed through any standard web browser by appending the LSID to the prefix “http://zoobank.org/”. The LSID for this publication is: urn:lsid:zoobank.org:pub:08C58B19-13BC-45CE-AEF5-BD1D508A1C10.

The online version of this work is archived via PubMed Central and LOCKSS and also available at http://www.zsm.mwn.de/mol/pub_schroedl.htm.

## Results

### Species description of *Pseudunela viatoris* sp. nov. from Fiji and Indonesia

#### Systematics

Family Pseudunelidae Rankin, 1979

Genus *Pseudunela* Salvini-Plawen, 1973

***Pseudunela viatoris*** sp. nov.

urn:lsid:zoobank.org:act: 9A559BA2-4EEE-4F3B-A1D2-A72ECB92096B.

Type Material—Holotype: ZSM Mol 20061954, stored in 75% EtOH; collected in Fiji, Viti Levu, Laucala Bay, Nukumbutho Island. GPS: 18°10.47′S, 178°28.34′E. Paratypes: ZSM Mol 20061945, 20 specimens stored in 75% EtOH; all paratypes collected together with holotype.

Etymology—*Pseudunela viatoris* sp. nov. is named after the latin word “viator” (engl. pilgrim/voyager) according to its supposed ability to travel over long distances.

Distribution—Known from Viti Levu, Fiji and Gili Lawa Laut, Indonesia.

In addition to the 3D plates please see also the supporting information (Fig S1): Interactive 3D-model of *Pseudunela viatoris* sp. nov. from Fiji.

#### External morphology

**Figure 1:**
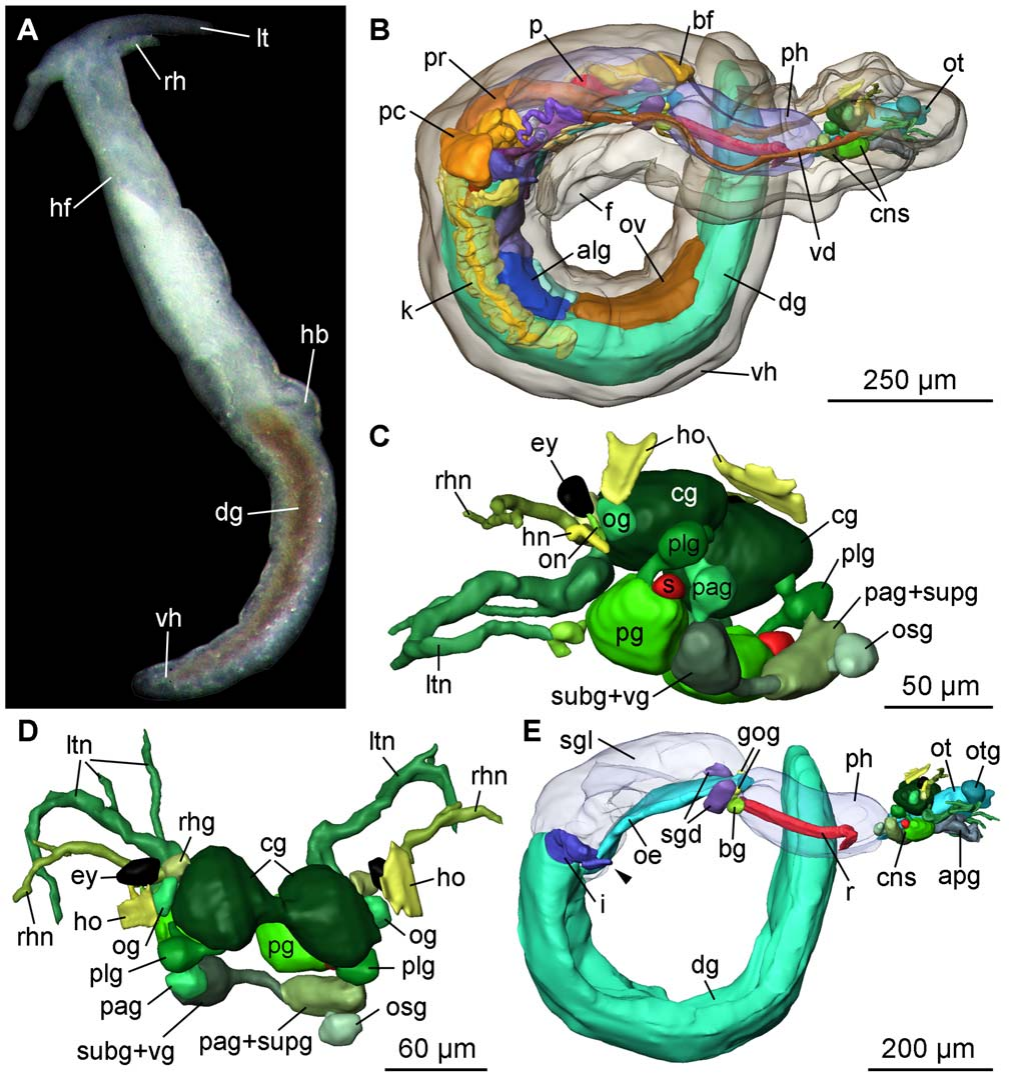
Photograph of a living specimen and 3D reconstruction of *P. viatoris* sp. nov. from Fiji. A: external morphology of a living specimen (body size 3 mm), dorsal view. B: general anatomy, right view. C: CNS, left view. D: CNS, dorsal view. E: digestive system with CNS, right view. Abbreviations: **alg**, albumen gland; **apg**, anterior pedal gland; **bf**, basal finger; **bg**, buccal ganglion; **cg**, cerebral ganglion; **cns**, central nervous system; **dg**, digestive gland; **ey**, eye; **f**, foot; **gog**, gastro-oesophageal ganglion; **hb**, heart bulb; **hf**, head-foot complex; **hn**, Hancock's nerve; **ho**, Hancock's organ; **i**, intestine; **k**, kidney; **lt**, labial tentacle; **ltn**, labial tentacle nerve; **oe**, oesophagus; **og**, optic ganglion; **on**, optic nerve; **osg**, osphradial ganglion; **ot**, oral tube; **otg**, oral tube gland; **ov**, ovotestis; **p**, penis; **pag**, parietal ganglion; **pc**, pericardium; **pg**, pedal ganglion; **ph**, pharynx; **plg**, pleural ganglion; **pr**, prostate; **r**, radula; **rh**, rhinophore; **rhg**, rhinophoral ganglion; **rhn**, rhinophoral nerve; **s**, statocyst; **sgd**, salivary gland duct; **sgl**, salivary gland; **subg**, subintestinal ganglion; **supg**, supraintestinal ganglion; **vd**, vas deferens; **vg**, visceral ganglion; **vh**, visceral hump; **arrowhead**, common opening of digestive and excretory systems. **The interactive 3D-model** of *P. viatoris* sp. nov. can be accessed by clicking onto the figure in the supporting information figure S1 (Adobe Reader Version 7 or higher required). Rotate model by dragging with left mouse button pressed, shift model: same action+ctrl (or change default action for left mouse button), zoom: use mouse wheel. Select or deselect (or change transparency of) components in the model tree, switch between prefab views or change surface visualization (e.g. lightning, render mode, crop etc.).

**Figure 2:**
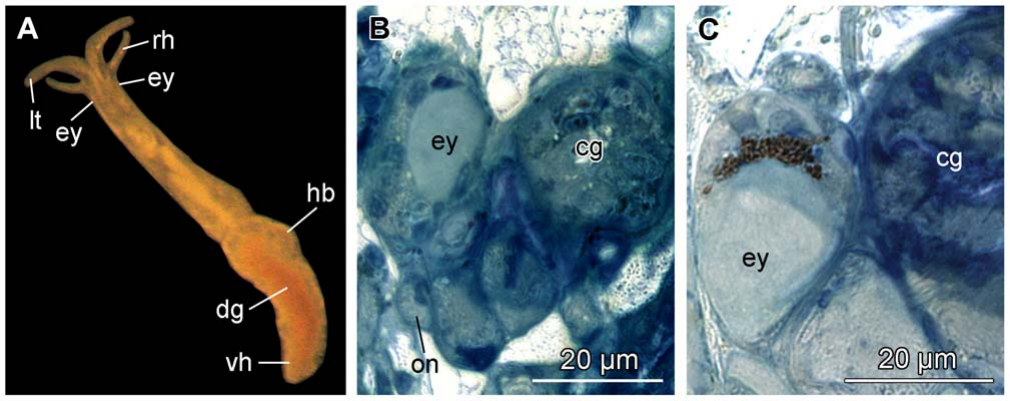
Photograph of a living specimen and histological cross-sections of *P. viatoris* sp. nov. from Indonesia. A: external morphology of a living specimen (body size 3 mm). B: unpigmented eye. C: pigmented eye. Abbreviations: **cg**, cerebral ganglion; **dg**, digestive gland; **ey**, eye; **hb**, heart bulb; **lt**, labial tentacle; **on**, optic nerve; **rh**, rhinophore; **vh**, visceral hump.

**Figure 3:**
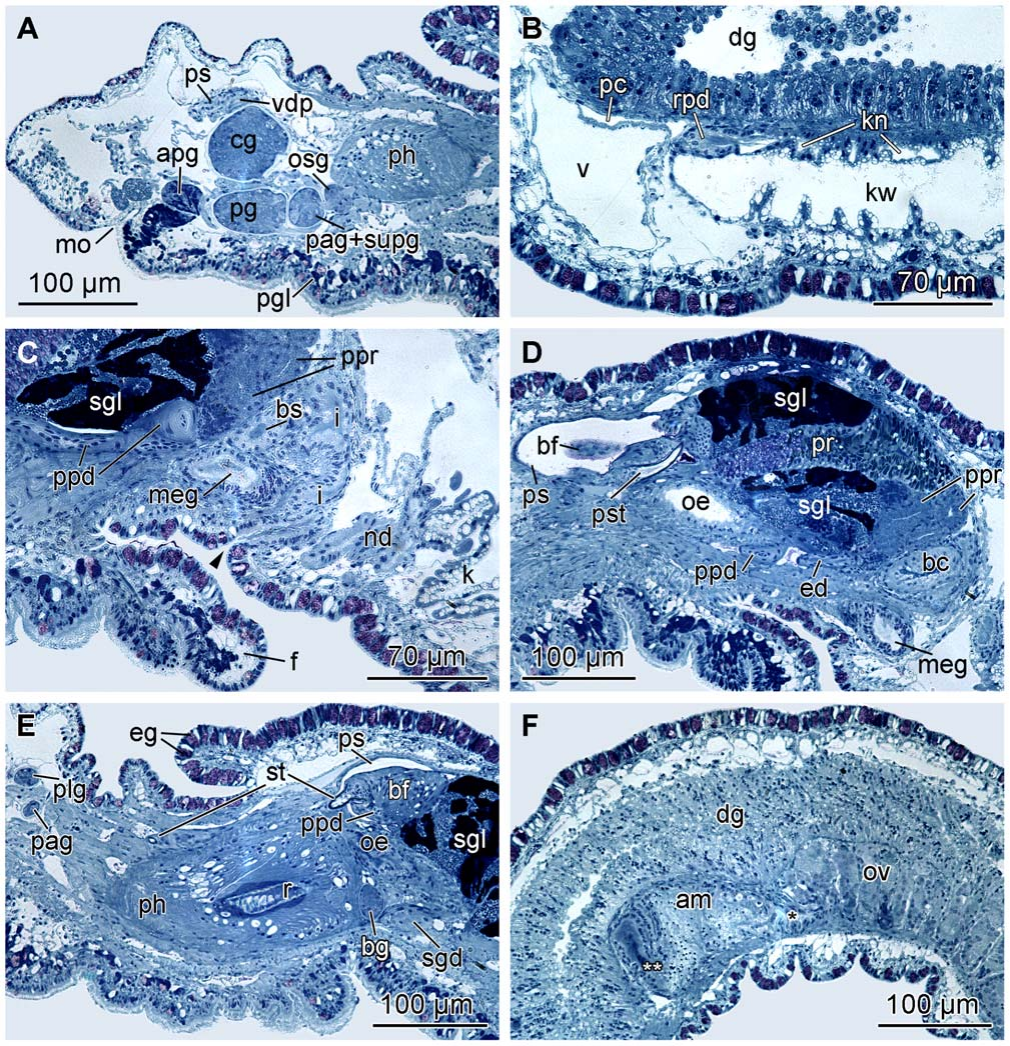
Histological cross-sections of *P. viatoris* sp. nov. from Fiji. A: anterior pedal gland and ganglia. B: circulatory and excretory systems. C: common opening of digestive and excretory systems. D: penial stylet and prostate. E: basal finger and pharynx. F: ampulla and ovotestis. Abbreviations: **am**, ampulla; **apg**, anterior pedal gland; **bc**, bursa copulatrix; **bf**, basal finger; **bg**, buccal ganglion; **bs**, bursa stalk; **cg**, cerebral ganglion; **dg**, digestive gland; **ed**, ejaculatory duct; **eg**, epidermal gland; **f**, foot; **i**, intestine; **k**, kidney; **kn**, narrow lumen of kidney; **kw**, wide lumen of kidney; **meg**, membrane gland; **mo**, mouth opening; **nd**, nephroduct; **oe**, oesophagus; **osg**, osphradial ganglion; **ov**, ovotestis; **pag**, parietal ganglion; **pc**, pericardium; **pg**, pedal ganglion; **pgl**, pedal gland; **ph**, pharynx; **plg**, pleural ganglion; **ppd**, paraprostatic duct; **ppr**, paraprostate; **pr**, prostate; **ps**, penial sheath; **pst**, penial stylet; **r**, radula; **rpd**, renopericardioduct; **sgd**, salivary gland duct; **sgl**, salivary gland; **st**, stylet of basal finger; **supg**, supraintestinal ganglion; **v**, ventricle; **vdp**, posterior-leading vas deferens; *, pre-ampullary gonoduct; **, post-ampullary gonoduct; **arrowhead**, common opening of digestive and excretory systems.

The body of *Pseudunela viatoris* sp. nov. is divided into an anterior head-foot complex (hf) and a posterior elongated visceral hump (vh) (Fig. 1A). The paired labial tentacles (lt) are broad at the base and taper to the end. The rhinophores (rh) are tapered and shorter and thinner than the labial tentacles (Fig. 1A). The densely ciliated foot (f) is as broad as the anterior head-foot complex and extends about one third of the elongated visceral hump (Fig. 1B). The heart bulb (hb) (Fig. 1A) is visible externally in the anterior part of the visceral hump on the right body side. Subepidermal, needle-shaped calcareous spicules are sparsely distributed in the cephalic tentacles, the foot and the visceral hump; in the anterior part of the latter they are larger than in the posterior part. The body colour is whitish translucent, the digestive gland (dg) (Fig. 1A) is brownish coloured (in specimens from Indonesia: orange-brownish (Fig. 2A)) shining through the epidermis. Epidermal glands (eg) (Fig. 3E) are distributed particularly over the visceral hump. The body size of living specimens is about 3 mm. Whereas eyes are not visible externally in specimens from Fiji (Fig. 1A), eyes (ey) are weakly visible in some specimens from Indonesia (Fig. 2A).

#### Microanatomy: Central nervous system (CNS)

**Figure 4:**
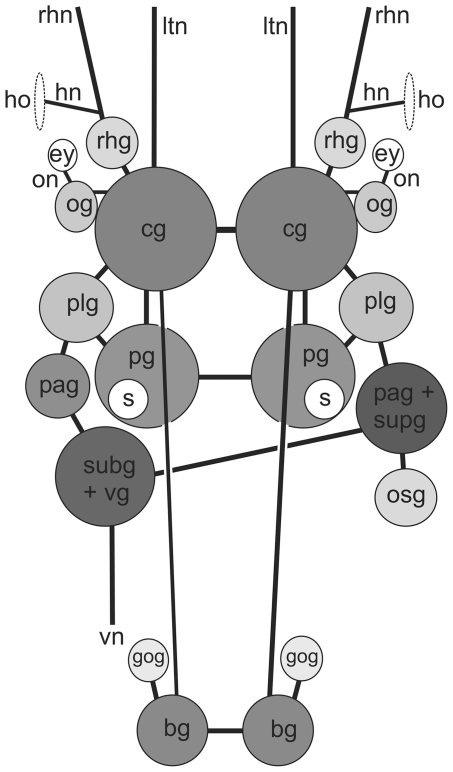
CNS of *P. viatoris* sp. nov. from Fiji (schematic overview, dorsal view). Abbreviations: **bg**, buccal ganglion; **cg**, cerebral ganglion; **ey**, eye; **gog**, gastro-oesophageal ganglion; **hn**, Hancock's nerve; **ho**, Hancock's organ; **ltn**, labial tentacle nerve; **og**, optic ganglion; **on**, optic nerve; **osg**, osphradial ganglion; **pag**, parietal ganglion; **pg**, pedal ganglion; **plg**, pleural ganglion; **rhg**, rhinophoral ganglion; **rhn**, rhinophoral nerve; **s**, statocyst; **subg**, subintestinal ganglion; **supg**, supraintestinal ganglion; **vg**, visceral ganglion; **vn**, visceral nerve. Not to scale.

The euthyneurous CNS of *Pseudunela viatoris* sp. nov. consists of the paired cerebral (cg), rhinophoral (rhg), optic (og), pedal (pg), pleural (plg), buccal (bg) and gastro-oesophageal ganglia (gog) and three distinct ganglia on the visceral nerve cord, plus an osphradial ganglion (osg) (Fig. 4). All ganglia excluding the buccal and gastro-oesophageal ganglia are located pre-pharyngeally (Fig. 1E). The cerebral, pedal and pleural ganglia are linked by short connectives forming the pre-pharyngeal nerve ring. The strong labiotentacular nerve (ltn) (Figs. 1C, D; 4) emerges from the cerebral ganglion innervating the labial tentacle. A rhinophoral ganglion (Figs. 1 D; 4) is connected anterodorsally to each cerebral ganglion by a short, single cerebro-rhinophoral connective. A nerve arises from the rhinophoral ganglion and bifurcates at its base. The rhinophoral nerve (rhn) (Figs. 1C, D; 4) innervates the rhinophore and the Hancock's nerve (hn) (Figs. 1C; 4) extends to the paired Hancock's organ (ho) (Figs. 1C, D; 4). The latter is a ciliated groove just behind the rhinophore. An optic ganglion (Figs. 1C, D; 4) is connected laterally to each cerebral ganglion by a thin nerve. The optic nerve (on) (Figs. 1C; 4) emerges from the optic ganglion innervating the unpigmented eye (ey) (Figs. 1C, D; 4) of 30–35 µm. In specimens from Indonesia unpigmented (Fig. 2B) and pigmented (Fig. 2C) eyes are present. Precerebral accessory ganglia are absent. The pedal commissure is slightly longer than the cerebral commissure. A statocyst (Figs. 1C; 4) is attached dorsally to each pedal ganglion. The pleural ganglia (Figs. 1C, D; 4) are connected by very short connectives to the visceral nerve cord, thus the latter is arranged anterior to the pharynx. There are three separate ganglia on the visceral nerve cord: the left parietal ganglion (pag), the fused subintestinal/visceral ganglion (subg+vg) and the fused right parietal/supraintestinal ganglion (pag+supg) (Figs. 1C, D; 4). Only the subintestinal/visceral-parietal/supraintestinal connective is long. An osphradial ganglion (Figs. 1C, D; 3A; 4) is connected to the fused parietal/supraintestinal ganglion. No histologically differentiated osphradium could be detected. The buccal ganglia (Figs. 1E; 3E; 4) are located posterior to the pharynx and the short buccal commissure runs ventrally to the oesophagus. A small gastro-oesophageal ganglion (Figs. 1E; 4) is connected dorsally to each buccal ganglion.

#### Microanatomy: Digestive system

**Figure 5:**
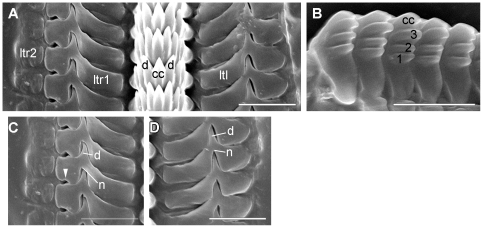
SEM micrographs of the radula of *P. viatoris* sp. nov. from Fiji. A: row of radular teeth. B: rhachidian tooth. C: right lateral teeth. D: left lateral tooth. Abbreviations: **cc**, central cusp; **d**, denticle; **ltl**, left lateral tooth; **ltr1**, first right lateral tooth; **ltr2**, second right lateral tooth; **n**, notch; **rh**, rhachidian tooth; **1**,**2**,**3**, lateral denticle on rhachidian tooth; **arrowhead**, emargination.

**Figure 6:**
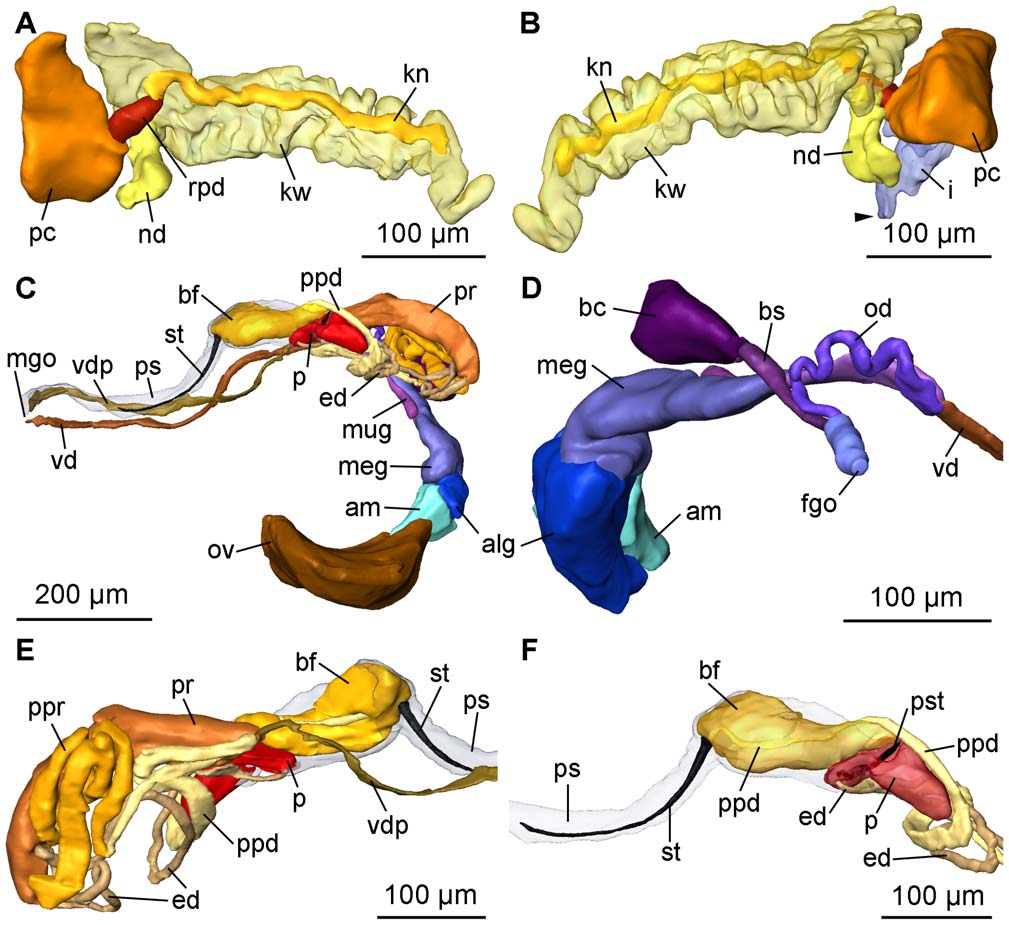
3D reconstruction of the excretory and reproductive systems of *P. viatoris* sp. nov. from Fiji. A: circulatory and excretory systems, left view. B: circulatory and excretory systems, right view. C: complete reproductive system, left view. D: nidamental glands and sperm storing receptacles, right view. E: anterior male copulatory organs, right view. F: penis and basal finger, left view. Abbreviations: **alg**, albumen gland; **am**, ampulla; **bc**, bursa copulatrix; **bf**, basal finger; **bs**, bursa stalk; **ed**, ejaculatory duct; **fgo**, female gonopore; **i**, intestine; **kn**, narrow lumen of kidney; **kw**, wide lumen of kidney; **meg**, membrane gland; **mgo**, male gonopore; **mug**, mucus gland; **nd**, nephroduct; **od**, oviduct; **ov**, ovotestis; **p**, penis; **pc**, pericardium; **ppd**, paraprostatic duct; **ppr**, paraprostate; **pr**, prostate; **ps**, penial sheath; **pst**, penial stylet; **rpd**, renopericardioduct; **st**, stylet of basal finger; **vd**, vas deferens; **vdp**, posterior-leading vas deferens; **arrowhead**, common opening of digestive and excretory systems.

The mouth opening (mo) (Fig. 3A) is situated ventrally between the labial tentacles. The paired anterior pedal glands (apg) (Figs. 1E; 3A) discharge ventrally of the mouth opening to the exterior. The oral tube (ot) (Fig. 1E) is long and flanked by paired oral tube glands (otg) (Fig. 1E) which discharge in its anterior part. The hook-shaped radula (r) (Figs. 1E; 3E) is approx. 180 µm long and embedded within the muscular pharynx (ph) (Figs. 1E; 3E). The radula formula is 44–50×1.1.2 with 32–37 teeth on the upper ramus and 12–17 teeth on the lower one. The triangular rhachidian tooth (Fig. 5B) bears one projecting central cusp (cc) with 3–4 lateral denticles (d) on each side. The first pair of lateral denticles shows almost the same size as the central cusp, the other denticles are smaller. The left lateral tooth (ltl) (Fig. 5A, D) is plate-like and has a well-developed, pointed denticle on their anterior margin and a prominent notch (n) on the posterior one, in which the denticle of the anterior lateral tooth matches. The right lateral teeth (ltr) (Fig. 5A, C) consist of two plates; the first inner one shows also a denticle on its anterior margin and a small emargination (Fig. 5C) next to the notch, the second outer lateral tooth lacks any denticle. The inner margins of the first lateral plates are always rounded; the outer margin of the left lateral tooth is rounded as well, whereas strait in the right lateral tooth. In the specimens from Indonesia the rhachidian tooth shows 2–4 denticles per side. The presence or absence of a second lateral tooth on the right side cannot be confirmed here; however, there is an emargination present and the outer margin of the first right lateral tooth is strait as in the Fijian specimens. These features may indicate a second lateral tooth in the specimen from Indonesia, as well. Jaws are absent. The oesophagus (oe) (Figs. 1E; 3D, E) is long and ciliated. In the anterior part one pair of large salivary glands (sgl) (Figs. 1E; 3C, D) is connected via salivary gland ducts (sgd) (Figs. 1E; 3E). The sac-like digestive gland (dg) (Figs. 1E; 3F) extends to the posterior end of the visceral hump (Fig. 1A, B). The intestine (i) (Figs. 1E; 3C) is densely ciliated and short. It receives the nephroduct (nd) before opening as a common duct (Figs. 3C; 6B) ventrolaterally on the right side of the visceral hump and posterior to the female gonopore to the exterior.

#### Microanatomy: Circulatory and excretory systems

**Figure 7:**
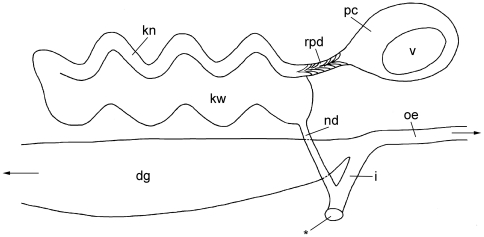
Circulatory and excretory systems of *P. viatoris* sp. nov. from Fiji (schematic drawing, right view). Abbreviations: **dg**, digestive gland; **i**, intestine; **kn**, narrow lumen of kidney; **kw**, wide lumen of kidney; **nd**, nephroduct; **oe**, oesophagus; **pc**, pericardium; **rpd**, renopericardioduct; **v**, ventricle; *, common opening of excretory and digestive systems. Drawing not to scale.

The circulatory and excretory systems are situated at the beginning of the visceral hump at the right side of the body (Fig. 1B). The circulatory system comprises a thin-walled pericardium (pc) (Figs. 6A, B; 7) surrounding a large one-chambered heart (v) (Figs. 3B; 7). The aorta could not be detected. The renopericardioduct (rpd) (Figs. 3B; 6A; 7) is a well-developed, densely ciliated funnel. The kidney (k) is an elongated sac (Fig. 1B) that extends over the anterior half of the visceral hump. Internally it is subdivided into two histologically distinct sections: a narrow lumen (kn) bordered by tissue with small vacuoles, and a wide lumen (kw) limited by tissue with large vacuoles (Figs. 3B; 6A, B; 7). The renopericardioduct connects to the excretory system in the anterior part of the kidney to its narrow lumen (Fig. 3B). The latter joins the wide lumen in the posterior part of the kidney (Fig. 7). The transition of the kidney and the nephroduct is narrow and ciliated. The nephroduct (Figs. 6A, B; 7) is short and empties into the distal part of the intestine just before the opening to the exterior (Figs. 3C; 7).

#### Microanatomy: Reproductive system

The terminology used below follows basically Ghiselin [39], Klussmann-Kolb [40] and Haase & Wawra [41].

**Figure 8:**
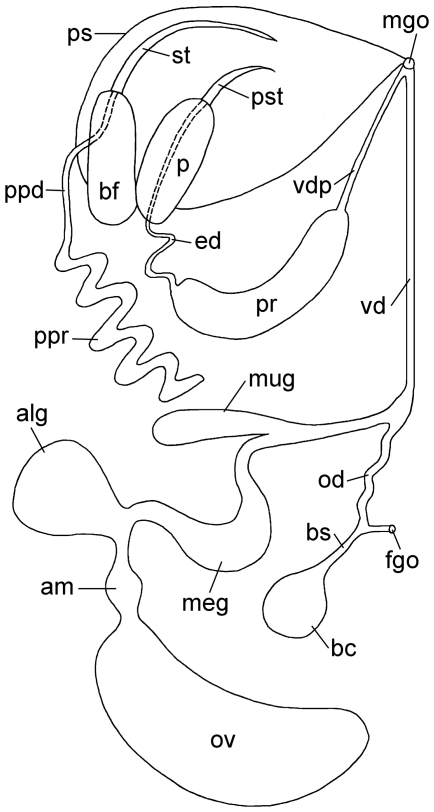
Reproductive system of *P. viatoris* sp. nov. from Fiji (schematic drawing, dorsal view). Abbreviations: **alg**, albumen gland; **am**, ampulla; **bc**, bursa copulatrix; **bf**, basal finger; **bs**, bursa stalk; **ed**, ejaculatory duct; **fgo**, female gonopore; **meg**, membrane gland; **mgo**, male gonopore; **mug**, mucus gland; **od**, oviduct; **ov**, ovotestis; **p**, penis; **ppd**, paraprostatic duct; **ppr**, paraprostate; **pr**, prostate; **ps**, penial sheath; **pst**, penial stylet; **st**, stylet of basal finger; **vd**, vas deferens; **vdp**, posterior-leading vas deferens. Not to scale.

Note: Morse [42] reported on a *Pseudunela* species from Fiji. However, at present stage of knowledge we would not like to assign her specimens to our species *P. viatoris* sp. nov. from Fiji. Due to a different collecting site in Morse [42] we cannot exclude that there are two different *Pseudunela* species on different Fijian islands. On the Solomon Islands we found two distinct species on the same island, at neighbouring beaches. Furthermore, Morse's drawing ([42] fig. 4A) indicates the presence of externally visible eyes which is definitely not applicable for our species. Nevertheless, there are pigmented and externally visible eyes in at least one specimen of *P. viatoris* sp. nov. from Indonesia, but our molecular results show great similarities even on the fast evolving mitochondrial markers, despite of the large geographic distance.

Specimens of *Pseudunela viatoris* sp. nov. have a hermaphroditic and special androdiaulic reproductive system. The sac-like ovotestis (ov) (Figs. 1B; 6C; 8) extends over the half of the visceral hump and is separated into follicles (Fig. 3F). No yolky oocytes are developed in the examined specimen. Anterior to the ovotestis there is a tubular ampulla (am) (Figs. 3F; 6C, D; 8) filled with autosperm lying in disorder. Sperm heads are short (Fig. 3F). A receptaculum seminis (rs) is absent or not developed in the examined specimen. Three nidamental glands (Figs. 6C, D; 8) can be distinguished from proximal to distal: the sac-like blue-stained albumen gland (alg), the tubular purple-stained membrane gland (meg) and the sac-like purple-stained mucus gland (mug). The distal part of the mucus gland runs to the right side of the body where the hermaphroditic duct bifurcates into the vas deferens (vd) and the highly undulated oviduct (od) (Figs. 6D; 8). The bursa stalk (bs) (Figs. 3C; 6D; 8) connects to the large bursa copulatrix (bc) (Figs. 3D; 6D; 8) the content of which is stained dark blue. The oviduct and the bursa stalk join to a common duct just before opening through the female gonopore (fgo) (Figs. 6D; 8) laterally at the right side of the visceral hump to the exterior. The female gonopore is situated considerably anterior to the common opening of the digestive and the excretory systems. The internal vas deferens (Fig. 8) extends subepidermally up to the right rhinophore connecting the posterior reproductive system to the anterior male copulatory organs (Fig. 6E). The posterior-leading vas deferens (vdp) (Figs. 6E; 8) joins the tubular prostate gland (pr) (Figs. 3D; 6E; 8). The long, coiled and muscular ejaculatory duct (ed) (Figs. 3D; 6E, F) arises from the prostate and discharges at the top of the penis (p) through a hollow penial stylet (pst) (Figs. 3D; 6F; 8) of approx. 70 µm length (125 µm in a specimen from Indonesia). The blind ending and highly coiled glandular paraprostate (ppr) (Figs. 3D; 6E; 8) is longer and thinner than the prostate. The paraprostatic duct (ppd) (Figs. 3C, D; 6E, F) connects the paraprostate with the muscular basal finger (bf) (Fig. 6E, F), which is united to the penial muscle mass at its base. It enters the basal finger approx. in the upper half of the muscle (Fig. 6F) and discharges terminally via a hollow curved stylet (st) (Figs. 3E; 6F; 8) of about 200 µm length (30 µm in a specimen from Indonesia). Both stylets can be somewhat retracted into the muscles. Parts of the penis and the basal finger are surrounded by a thin-walled penial sheath (ps) (Figs. 3D; 6F; 8).

### Species description of *Pseudunela marteli* sp. nov. from the Solomon Islands and Vanuatu

#### Systematics

***Pseudunela marteli*** sp. nov.

urn:lsid:zoobank.org:act:77053243-8F24-4ED9-89DC-D5665814E750

Type Material—Holotype: ZSM Mol 20071803, stored in 99% EtOH; collected in Solomon Islands, Guadalcanal, Honiara, beach of “Art Gallery”. Paratypes: ZSM Mol 20090418, two specimens stored in 99% EtOH; ZSM Mol 20071851 (one serially sectioned specimen); all paratypes collected together with holotype.

Etymology—*Pseudunela marteli* sp. nov. with its large heart-bulb, is named in honour of our big-hearted friend and colleague Martin “Martl” Heß.

Distribution—Known from Guadalcanal, Solomon Islands and Oyster Island, Vanuatu.

#### Species diagnosis

External morphology and anatomy as in *P. viatoris* sp. nov. from Fiji.

#### Exceptions

**Figure 9:**
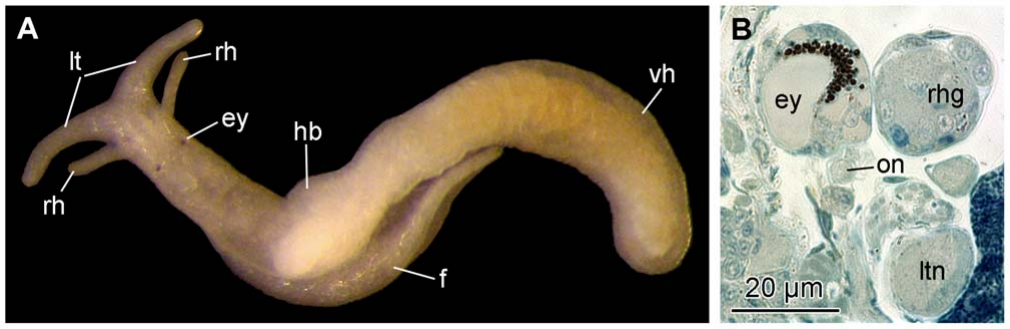
Photograph of a living specimen and histological cross-section of *P. marteli* sp. nov. (Solomon Islands). A: external morphology of a living specimen (body size 3 mm). B: pigmented eye. Abbreviations: **ey**, eye; **f**, foot; **hb**, heart bulb; **lt**, labial tentacle; **ltn**, labial tentacle nerve; **on**, optic nerve; **rh**, rhinophore; **rhg**, rhinophoral ganglion; **vh**, visceral hump.

Note: Specimens of *P. marteli* sp. nov. collected in Vanuatu (Fig. 10) differ from those collected on the Solomon Islands in some details: the pigmented eyes are slightly smaller (25–30 µm) and only weakly visible externally (Fig. 10A); subepidermal spicules are situated additionally around the CNS (Fig. 10D); the hollow curved penial stylet is longer measuring 180–200 µm in length; the ampulla (Fig. 10F) is tubular; the albumen and the mucus glands (Fig. 10E) are sac-like, the membrane gland (Fig. 10F) is tubular. Based on these anatomical differences both populations could, however, not satisfyingly be delimited due to potential intraspecific variation (see discussion). Future comparative analyses dedicated to evaluate the degree of intraspecific variation might, however, lead to a delineation of both populations.

Colour of digestive gland greenish or orange-brownish (Fig. 9A); eyes (30–35 µm) pigmented (Fig. 9B) and well visible externally (Fig. 9A); foot length up to half of the visceral hump (Fig. 9A); subepidermal spicules more abundant in cephalic tentacles, foot and visceral hump. The radula formula is 57–59×1.1.?; rhachidian tooth with 3–4 denticles per side. The hollow curved penial stylet measures 130 µm in length, the stylet of basal finger is 30 µm long. The ampulla is sac-like; allosperm receptacles are absent in the examined specimen. The albumen and mucus glands are tubular; the membrane gland is sac-like.

**Figure 10:**
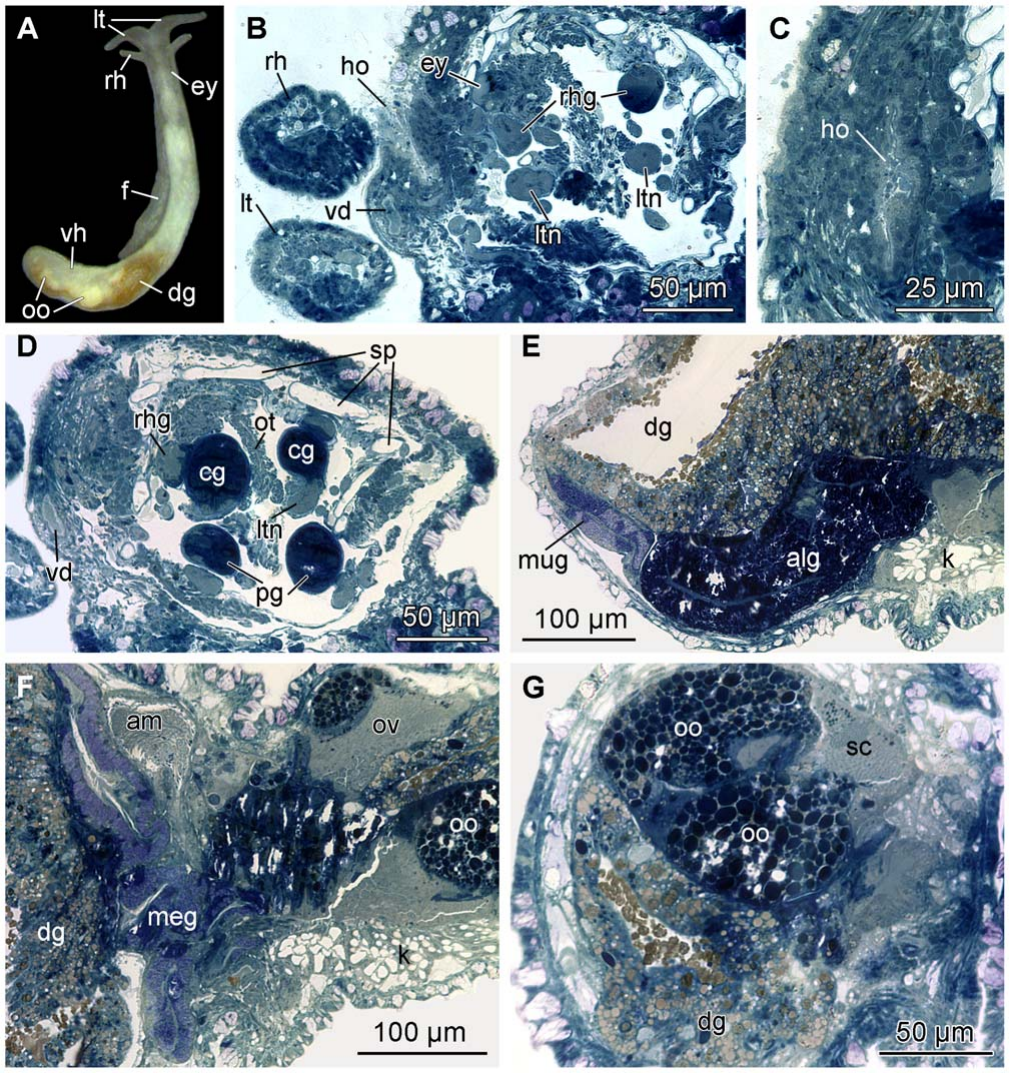
Histological cross-sections of *P. marteli* sp. nov. from Vanuatu. A: external morphology of a living specimen (body size 3 mm). B: Hancock's organ and eye. C: Hancock's organ. D: spicule cavities. E: albumen and mucus glands. F: ampulla and membrane gland. G: oocytes and spermatocytes. Abbreviations: **alg**, albumen gland; **am**, ampulla; **cg**, cerebral ganglion; **dg**, digestive gland; **ey**, eye; **f**, foot; **ho**, Hancock's organ; **k**, kidney; **lt**, labial tentacle; **ltn**, labial tentacle nerve; **meg**, membrane gland; **mug**, mucus gland; **oo**, oocyte; **ot**, oral tube; **ov**, ovotestis; **pg**, pedal ganglion; **rh**, rhinophore; **rhg**, rhinophoral ganglion; **sc**, spermatocytes; **sp**, spicule cavity; **vd**, vas deferens; **vh**, visceral hump.

### Molecular results

**Figure 11:**
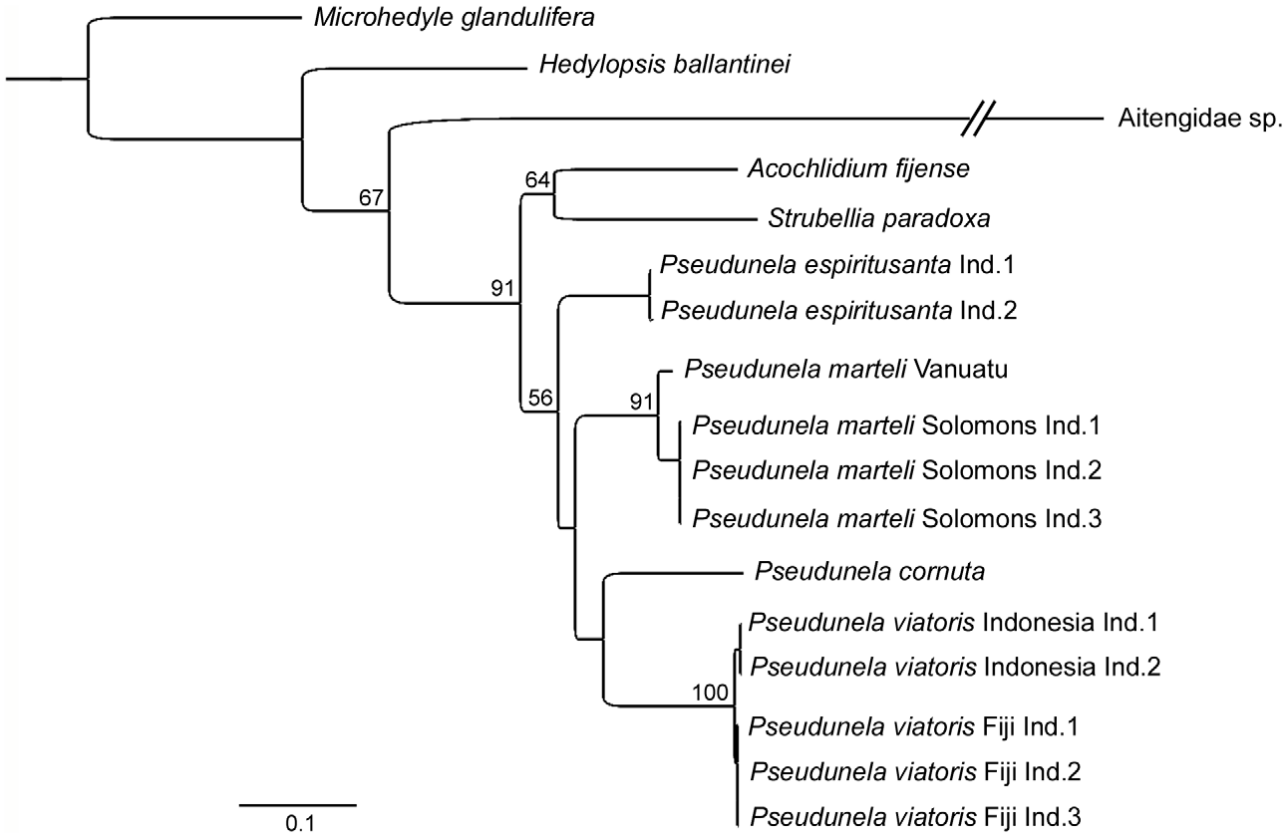
Molecular phylogeny of the genus *Pseudunela*. RAxML analysis of concatenated sequences of partial 18S rRNA, 16S rRNA and COI markers, analysed in four partitions. Bootstrap values (>50%) given at nodes. Sister group relationship between Pseudunelidae and limnic Acochlidiidae receives strong support. Within *Pseudunela*, brackish *P. espiritusanta* is basal to the remaining species, but sister group relationships within *Pseudunela* do not gather any bootstrap support.

The result of the maximum likelihood analysis of the concatenated dataset analysed in four partitions is shown in Fig. 11. The genus *Pseudunela* results monophyletic, but with low support (bootstrap value (BS) 56%). The sister group relationship of *Pseudunela* (i.e. Pseudunelidae) with limnic Acochlidiidae is well supported (BS 91%). The internal phylogeny of *Pseudunela* is fully resolved, but the sister group relationships within the genus do not gather support. All morphologically defined *Pseudunela* lineages are recovered as monophyletic. The topological species delimitation based on the available molecular dataset (combining nuclear and mitochondrial markers) results in four different clades within the genus *Pseudunela*, supporting the morphological descriptions of *P. viatoris* and *P. marteli* spp. nov..

Pairwise genetic differences and values of intraspecific variation were generated based on partial mitochondrial COI and 16S rRNA using Species Identifier. The largest variation within the different populations of *Pseudunela* species is relatively low (0.15–0.45% on partial COI and 0.0–0.69% on partial 16S rRNA). The largest intraspecific uncorrected p-distances among *P. viatoris* sp. nov. are 1.67% on COI and 1.39% on 16S rRNA (n = 5), in *P. marteli* sp. nov. the largest distance between individuals of Solomon Island and Vanuatu populations is comparably high with 5.49% on COI and 3.24% on 16S rRNA. Between species, the smallest interspecific distances within *Pseudunela* were considerably larger with 14.04–16.48% on COI and 8.82–14.85% on 16S rRNA; smallest interspecific distances occurred between the morphologically clearly distinct *P. espiritusanta* and *P. marteli* sp. nov. (see Tables 3, 4, 5, 6).

**Table 3:** Comparison of the external morphology within the genus *Pseudunela*.

	*P. espiritusanta* Neusser & Schrödl, 2009	*P. cornuta* (Challis, 1970)	*P. eirene* (Wawra, 1988)	*Pseudunela viatoris* sp. nov.	*Pseudunela viatoris* sp. nov.	*Pseudunela marteli* sp. nov.	*Pseudunela marteli* sp. nov.
Collection site	Espiritu Santo, Vanuatu	Guadalcanal, Solomon Islands	Andaman Islands, India	Viti Levu, Fiji	Gili Lawa Laut, Indondesia	Guadalcanal, Solomon Islands	Espiritu Santo, Vanuatu
Data source	Neusser & Schrödl 2009	Challis 1970; **Neusser et al. 2009**	Wawra 1988	present study	present study	present study	present study
Habitat	brackish	marine; *	marine	marine	marine	marine	marine
Body size (mm)	9	3 ; *	4 (fixed specimen)	3	3–4	3	3
Colour of body	translucent-whitish	translucent-whitish; *	?	translucent-whitish	translucent-whitish	translucent-whitish	translucent-whitish
Colour of digestive gland	yellowish	?; **orange-brownish**	?	brownish	orange-brownish	greenish or orange-brownish	orange-brownish
Eyes visible externally	well	no; *	?	no	weakly	well	weakly
Foot width	broader than body	as broad as head; *	as broad as body	as broad as body	as broad as body	as broad as body	as broad as body
Foot length	2/3 of vh	slightly longer than anterior body; **1/2 of vh**	?	1/3 to 1/2 of vh	1/3 to 1/2 of vh	1/2 of vh	1/2 of vh
Visceral hump	bent, recurved	elongated; *	?	elongated	elongated	elongated	elongated
Heart bulb visible	yes	?; **yes**	?	yes	yes	yes	yes
Subepidermal calcareous spicules	bean-shaped; in cephalic tentacles, foot, vh, around CNS	absent; **few in vh**	?	in cephalic tentacles, foot and vh	in cephalic tentacles, foot and vh	in cephalic tentacles, foot, vh,	in cephalic tentacles, foot, vh, around CNS

**CNS**, central nervous system; **vh**, visceral hump; **?**, no data available; revised data in **bold**, * = confirmed.

**Table 4:** Comparison of the central nervous system and the radula within the genus *Pseudunela*.

	*P. espiritusanta* Neusser & Schrödl, 2009	*P. cornuta* (Challis, 1970)	*P. eirene* (Wawra, 1988)	*Pseudunela viatoris* sp. nov.	*Pseudunela viatoris* sp. nov.	*Pseudunela marteli* sp. nov.	*Pseudunela marteli* sp. nov.
Collection site	Espiritu Santo, Vanuatu	Guadalcanal, Solomon Islands	Andaman Islands, India	Viti Levu, Fiji	Gili Lawa Laut, Indondesia	Guadalcanal, Solomon Islands	Espiritu Santo, Vanuatu
Data source	Neusser & Schrödl 2009	Challis 1970; **Neusser et al. 2009**	Wawra 1988	present study	present study	present study	present study
Accessory ganglia	absent	present; **absent**	present	absent	absent	absent	absent
Optic ganglion	present	absent; **present**	?	present	present	present	present
Origin of optic nerve	optic ganglion	?; **rhinophoral nerve**	?	optic ganglion	optic ganglion	optic ganglion	optic ganglion
Eye pigment	present	?; **absent**	?	absent	absent/present	present	present
Eye diameter (µm)	45	?; **20**	?	30–35	30–35	30–35	25–30
Hancock's organ	present	?; **?**	?	present	?	present	present
Osphradial ganglion	present	absent; **present**	present	present	present	present	present
Gastro-oesophageal ganglion	present	absent; **present**	absent	present	?	present	present
Radula formula	67×1.1.2	50×1.1.1; **?**	52×1.1.2	44–50×1.1.2	38×1.1.?	57–59×1.1.?	57×?
Rhachidian cusp	projecting	projecting; **?**	?	projecting	projecting	projecting	projecting
Rhachidian tooth denticles/side	4–7	3–4; **?**	3–4	3–4	2–4	3–4	3–4

**?**, no data available; revised data in **bold**.

**Table 5:** Comparison of the circulatory and excretory systems within the genus *Pseudunela*.

	*P. espiritusanta* Neusser & Schrödl, 2009	*P. cornuta* (Challis, 1970)	*P. eirene* (Wawra, 1988)	*Pseudunela viatoris* sp. nov.	*Pseudunela viatoris* sp. nov.	*Pseudunela marteli* sp. nov.	*Pseudunela marteli* sp. nov.
Collection site	Espiritu Santo, Vanuatu	Guadalcanal, Solomon Islands	Andaman Islands, India	Viti Levu, Fiji	Gili Lawa Laut, Indondesia	Guadalcanal, Solomon Islands	Espiritu Santo, Vanuatu
Data source	Neusser & Schrödl 2009	Challis 1970; **Neusser et al. 2009**	Wawra 1988	present study	present study	present study	present study
Anal-genital cloaca	absent	present; **absent**	?	absent	absent	absent	absent
Common opening of digestive and excretory system (a/np)	absent	absent; *	?	present	present	present	present
Heart	ventricle	ventricle; **atrium and ventricle**	?	ventricle	ventricle	ventricle	ventricle
Renopericardioduct	long, ciliated funnel	present; **long, ciliated funnel**	?	long, ciliated funnel	long, ciliated funnel	long, ciliated funnel	long, ciliated funnel
Kidney	long, internally divided	large, unfolded sac; **long, internally divided**	?	long, internally divided	long, internally divided	long, internally divided	long, internally divided
Nephroduct	long with two branches	?; **long with two branches**	?	short	short	short	short

**?**, no data available; revised data in **bold**, * = confirmed.

**Table 6:** Comparison of the reproductive system within the genus *Pseudunela*.

	*P. espiritusanta* Neusser & Schrödl, 2009	*P. cornuta* (Challis, 1970)	*P. eirene* (Wawra, 1988)	*Pseudunela viatoris* sp. nov.	*Pseudunela viatoris* sp. nov.	*Pseudunela marteli* sp. nov.	*Pseudunela marteli* sp. nov.
Collection site	Espiritu Santo, Vanuatu	Guadalcanal, Solomon Islands	Andaman Islands, India	Viti Levu, Fiji	Gili Lawa Laut, Indondesia	Guadalcanal, Solomon Islands	Espiritu Santo, Vanuatu
Data source	Neusser & Schrödl 2009	Challis 1970; **Neusser et al. 2009**	Wawra 1988	present study	present study	present study	present study
Hollow curved penial stylet (µm)	80	100 ; **600 (coiled 1.5 spirals)**	200	70	125	130	180–200
Solid basal thorn (µm)	absent	absent; *	30	absent	absent	absent	absent
Hollow curved stylet on basal finger (µm)	340	absent; **110**	?	200	30	30	30
Glands associated with copulatory organs	prostate, paraprostate	prostate, penial gland; **prostate, paraprostate**	?	prostate, paraprostate	prostate, paraprostate	prostate, paraprostate	prostate, paraprostate
Yolky oocytes developed	present	present; *	?	absent	?	absent	present
Ampulla	sac-like	?; **sac-like**	?	tubular	?	sac-like	tubular
Receptaculum seminis	present	?; **present**	?	absent	?	absent	absent
Bursa copulatrix	present	present; *	?	present	?	absent	absent
Albumen gland	tubular	?; tubular	?	sac-like	?	tubular	sac-like
Membrane gland	tubular	?; **tubular**		tubular		sac-like	tubular
Mucus gland	sac-like	sac-like	?	sac-like	?	tubular	sac-like

**?**, no data available; revised data in **bold**, * = confirmed.

Statistical parsimony analyses in TCS 1.21 of each mitochondrial marker (COI and 16S rRNA) congruently produce unconnected haplotype networks (not shown) for each of the herein morphologically defined *Pseudunela* species (i.e. *P. cornuta*, *P. espiritusanta*, *P. viatoris* sp. nov. (uniting populations from Fiji and Indonesia) and *P. marteli* sp. nov.). Moreover, the haplotype of *P. marteli* sp. nov. from Vanuatu is unconnected to the haplotypes from the Solomon population in both markers.

As an additional method of species delineation we applied GMYC to our molecular dataset, using a RAxML starting tree generated from the concatenated mitochondrial dataset (COI+16S). Under the multiple threshold option, GMYC recovers four entities, representing the above morphologically distinguished species: *P. cornuta*, *P. espiritusanta*, *P. marteli* sp. nov. and *P. viatoris* sp. nov.

## Discussion

### Morphology-based taxonomy

The *Pseudunela* specimens from different Indo-Pacific islands examined herein are compared according to their external morphology, microanatomy, and molecular markers. Externally, only the larger, recently discovered *Pseudunela espiritusanta* from Vanuatu [43] can be clearly distinguished from congeners by its much larger body size, the foot width and the shape of the visceral hump, as well as its unique brackish-water habitat (Table 3). In contrast, the herein examined, fully marine *Pseudunela* species all resemble externally *P. cornuta* from the Solomon Islands which was recently re-examined by Neusser et al. [17]. The body size and colour, the foot length and width, as well as the presence of subepidermal spicules do not differ between the species (Table 3). Only the visibility of the eyes through the body integument greatly varies among - and partly within - the marine *Pseudunela* species. In contrast to external features, our detailed anatomical examinations enable the discrimination of *P. cornuta* from the remaining marine *Pseudunela* species. Differences are related to all organ systems (Tables 4, 5, 6). The eyes are unpigmented and considerably smaller in *P. cornuta* than in the other *Pseudunela* species and they are not innervated by the optic ganglion, but the optic nerve emerges from the rhinophoral nerve [17]. The common opening of the excretory and digestive systems is absent in *P. cornuta* and the brackish-water *P. espiritusanta* [17], [43] and the anus and the nephropore open separately to the exterior. The most surprising feature concerns the excretory system with a complex kidney and a long, looped nephroduct consisting of two branches in *P. cornuta*. This kind of excretory system is characteristic for the brackish *P. espiritusanta* [43] and other limnic acochlidians studied in detail [44], [45]. In contrast, all marine *Pseudunela* species examined herein (i.e. *P. viatoris* and *P. marteli* spp. nov.) show a complex kidney as well, but have a short nephroduct as characteristic for other marine acochlidian species. Peculiar is the very long (600 µm) and curled, hollow penial stylet in *P. cornuta*, whereas the penial stylet in the other *Pseudunela* species is slightly curved but not curled and does not exceed 200 µm of length. The remaining *Pseudunela* species show several anatomical differences (mainly concerning the length of the copulatory stylets, and the shape of the ampulla and of the female glands; Table 6), which can be used for species delimitation. Such features, however, may depend on reproductive maturity and are not well explored yet. In summary, morphology-based taxonomy and even sophisticated 3D modelling of anatomical details as applied herein can only reveal parts of the actual species diversity of *Pseudunela* unambiguously; diagnosable microanatomical units found need to be tested by molecular phylogenetic analyses.

### Cryptic species?

The present molecular dataset is limited due to the low amount of individuals sampled, thus not allowing population genetic approaches and in depth comparison between intraspecific versus interspecific variation justifying molecularly based species delineation. Still, there are several lines of evidence supporting the defined microanatomical units as genetically separated partially cryptic lineages: 1) our maximum likelihood analyses based on a concatenated molecular dataset (combining nuclear and mitochondrial markers) recovers all microanatomical units as monophyla (Fig. 11). In our phylogenetic hypothesis *P. cornuta* separates cryptic *P. marteli* sp. nov. and *P. viatoris* sp. nov. 2) In contrast to earlier approaches relying on thresholds of divergence for the barcoding marker COI in molluscs [6], [21], [46], several recent studies showed that there is no universal threshold and that rates of intraspecific variation can outnumber supposedly ‘high’ rates of interspecific variation [34], [47]. Our limited dataset shows low rates of intraspecific variation, even when comparing far distant populations of *P. viatoris* sp. nov. from Fiji and Indonesia (n = 5; largest p-distance: 1.67% on partial COI, 1.39% on 16S rRNA). Then again interspecific variation among the microanatomically defined units is comparably high (14.04–16.48% on COI and 8.82–14.85% on 16S RNA) and the distances between the morphologically cryptic species are in the same range as to the morphologically clearly distinct *P. espiritusanta*. 3) In addition to ML tree-based methods and the comparison of pairwise distances, we generated haplotype networks applying 95% parsimony criterion, which resulted in unconnected haplotype networks for the described microanatomical units on both markers. Additionally, the *P. marteli* sp. nov. from Vanuatu (n = 1) is unconnected to the haplotype network of *P. marteli* sp. nov. from the Solomon Islands (n = 3) on both mitochondrial markers. 4) GMYC recovers all four microanatomical units; however, the performance and accuracy of GMYC to our knowledge has never been tested on such a small dataset, as ours. These independent molecular approaches are in congruence with our microanatomical units and thus, in our opinion, justify a separation in two formal new species.

There are several microanatomical differences between the two populations of *P. marteli* sp. nov. (e.g. size of eyes, length of penial stylet, see Tables 4, 5, 6), but intraspecific variation of these characters cannot be evaluated at present stage of knowledge and results from molecular data are incongruent (e.g. unconnected haplotype networks vs. one entity in GMYC). Moreover, the genetic distance between the two populations is low compared to the distances present in the closely related *Pseudunela* species. More data is needed to evaluate intraspecific variation and test conspecifity of the two *P. marteli* populations. Within specimens of *Pseudunela viatoris* sp. nov. from Fiji and Indonesia there are slight differences concerning the eye visibility and the length of stylets on the penial papilla, while stylets on the basal finger are remarkably different-sized. Specimens from Indonesia and Fiji cluster on different clades (Fig. 11). However, the genetic similarity between these specimens is very high (approx. 98–99% on COI and 16S rRNA) and intrapopulation variation is low. Thus, we do not consider these lineages to be specifically distinct, despite the distant geographic localities. More specimens are needed to explore morphological variability and genetic structure of these populations.

We conclude that we discovered morphologically cryptic species within the genus *Pseudunela*. External morphological, microanatomical and genetic evidences for recognizing species are congruent, and a combined approach of 3D-microanatomy and genetic markers can reliably distinguish and delineate all of the four species. Surprisingly, far distant geographic populations of specimens with slightly differing anatomy and presumably poor dispersive ability do not necessarily indicate different species, as revealed by highly similar mitochondrial sequences in *P. viatoris* sp. nov.. An integrative taxonomic approach combining morphological, 3D-microanatomical and molecular markers, like demonstrated here for *Pseudunela* species, thus is a powerful tool to independent structural or genetic approaches.

Overall, our results might be indicative for a still unknown diversity within mesopsammic gastropods. Recent studies on cryptic speciation within Meiofauna across taxa, has often revealed formerly considered wide-spread or even cosmopolitan species as flock of cryptic species (e.g. in proseriate flatworms [48], [49], polychaete annelids [50], [51] and gastrotrichs [52], [53]). Leading to the assumption that especially within this habitat, which is generally known for taxa with low dispersal abilities, there might be a high degree of cryptic speciation and the contribution of Meiofauna to marine biodiversity might be currently seriously underestimated [49]. However, some studies supported the presence of truly amphi-atlantic or cosmopolitan meiofaunal taxa, with the distribution and genetic interaction across Oceans in the absence of pelagic larvae still to be explained [50], [54].

### Distribution

The distribution of the four different *Pseudunela* species (*P. eirene* from Andaman Islands is not considered in this discussion as there exist only inadequate data and no material is available for detailed study) on the Indo-Pacific islands raises questions: 1) How can two different, genetically isolated *Pseudunela* species inhabit nearby beaches on one island with continuous coastline and 2) how can we explain the occurrence of *P. viatoris* sp. nov. on two far distant islands?

Considering that all Hedylopsacea occur in warm or tropical waters (except of *Hedylopsis spiculifera*, which inhabits temperate waters), we can assume that the common ancestor of the Pseudunelidae and Acochlidiidae s.l. has its origin in warm tropical waters as well. Recently, Jörger et al. [28] calibrated a molecular clock estimating divergence times for shell-less, and hence fossil-lacking Heterobranchia. In this study the origin of Acochlidia was estimated to the Mesozoic Triassic or Jurassic. According to the authors, the major diversification of Acochlidia took place in Jurassic, but the split between Pseudunelidae and Acochlidiidae was estimated to the Palaeogene. Even though this is a very rough estimation, it indicates that the diversification and distribution of the genus *Pseudunela* might have started over 35 mya, a long timeframe for a long-distance distribution, even for marine meiofaunal acochlidian species, which are regarded as poor dispersers. The hedylopsacean species *Pseudunela cornuta* [17] and *P. marteli* sp. nov. from Vanuatu, as well as the microhedylacean species, such as *Microhedyle remanei* (Marcus, 1953), *M. nahantensis* (Doe, 1974), *Parhedyle cryptophthalma* (Westheide & Wawra, 1974) and *Asperspina murmanica* (Kudinskaya & Minichev, 1978) [14], [55], [56], [57] have only a small number of large, yolky oocytes indicating a low reproductive output and a lecithotrophic development within a capsule rather than a planktotrophic larval development [10], [57]. Therefore, the distribution of larval and adult stages is expected only within a small radius step by step. Natural disasters (such as volcanic eruptions, earthquakes, heavy storms or erosion) or settlement by humans may disturb or even destroy sandy beaches [42]. This might result in genetically isolated populations or even local extinctions, which can explain the co-occurrence of two distinct *Pseudunela* species on nearby beaches. Another explication may be the adaptation to diverse, but subtle ecological conditions in the habitat, such as different currents, grain size, freshwater influx or food resources, which finally might result in separation of species.

The extensive distribution of *P. viatoris* sp. nov. is surprising. Due to aforementioned reasons a distribution of larvae via water currents is not likely. An accidentally distribution of different ontogenetic stages after heavy (sub-)tropical storms is not very probable due to the large distances. We cannot exclude a man-made dispersal, where small patches of sand of neighboured populations were displaced e.g. by ships. More likely, however, there exist intermediate populations between those from Fiji and Indonesia that have not been discovered yet – or already got extinct. Missing intermediates and restricted gene flow across these stepping stones might also explain the slight anatomical differences between the Fijian specimens and those from Indonesia, such as the variation in the length of the copulatory stylets or the pigmentation of the eye. Possibly, small genetic distances observed between these distant populations also may reflect a stage of ongoing allopatric speciation. Finally, another aspect should be considered: juveniles of the amphidromous nerite snail *Neritina asperulata* Recluz, 1842 show a “hitchhiking” behaviour by attaching to the shell of the congeneric *N. pulligera* Linnaeus, 1758. In this way young specimens travel upstream for growth and reproduction [58]. We can imagine that eggs and accordingly larval or adult acochlidians stick to e.g. benthic living organisms when the living conditions in the sand are changing for the worse and thus, may be displaced into another habitat [45].

### Phylogeny and evolution

Our molecular analysis (see Fig. 11) shows the marine and brackish-water *Pseudunela* as the sister group to the limnic Acochlidiidae s.l. and supports herein the results of recent morphological analysis [18] and previous molecular analysis [28]. Again, Aitengidae sp. clusters within the Hedylopsacea, as sister to Pseudunelidae plus Acochlidiidae [59]. The relationships between the *Pseudunela* species are fully resolved but with no robust support. As suspected by Neusser & Schrödl [43], the brackish *Pseudunela espiritusanta* from Vanuatu is the most basal *Pseudunela* species forming the sister group to all marine and temporary brackish *Pseudunela* species. The fully marine *P. marteli* sp. nov. from the Solomon Islands and Vanuatu form the sister group to the temporary brackish *P. cornuta* (also from the Solomon Islands) and the marine *P. viatoris* sp. nov. from Fiji and Indonesia. This tree topology (Fig. 11), however, does not clearly support previous ideas [18], i.e. that evolution within acochlidians was directed from marine to limnic habitats, possibly via brackish water. Instead, the ancestor of *Pseudunela* plus Acochlidiidae might have been already limnic or brackish water associated, with marine species evolving secondarily within *Pseudunela*.

**Figure 12:**
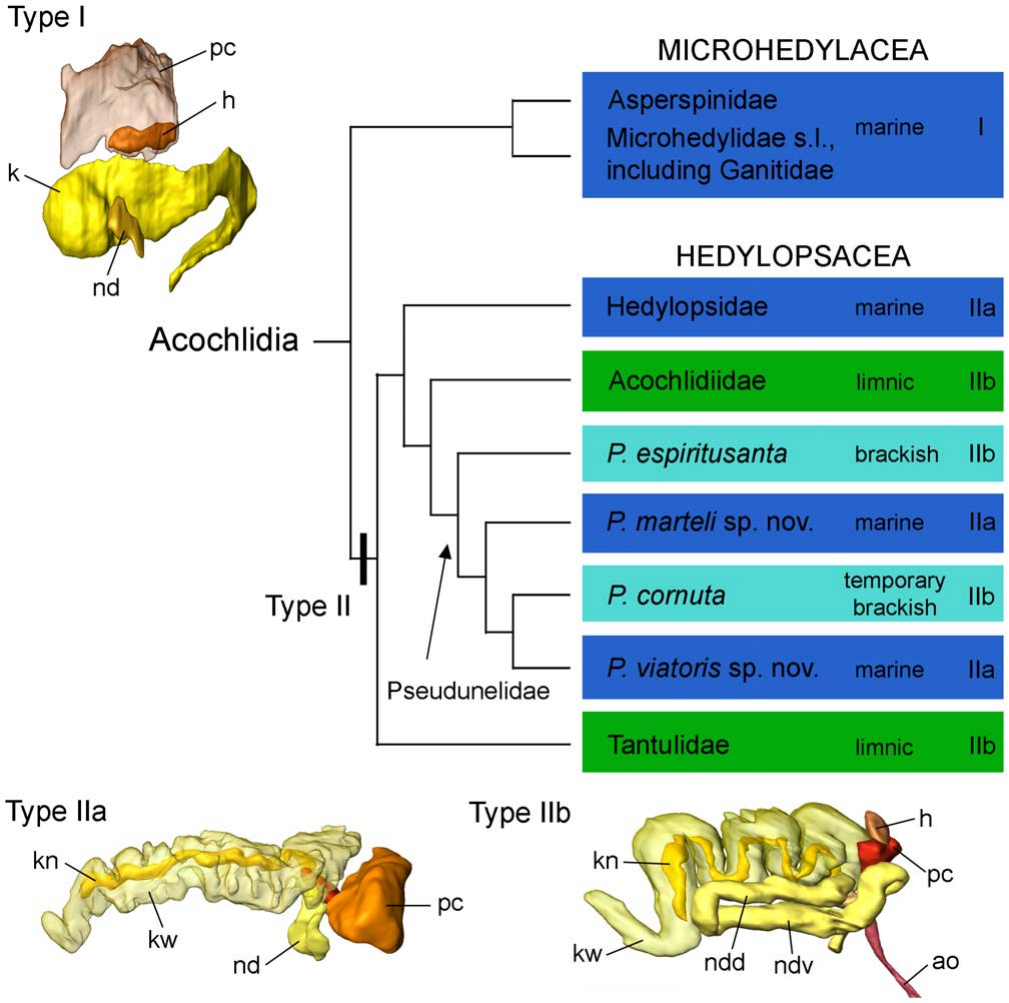
Evolution of excretory systems and habitat in acochlidian lineages. The habitat of the different acochlidian lineages and their types of excretory systems are plotted on a consensus tree (topology combined from Schrödl & Neusser [18] and molecular results herein; the enigmatic Aitengidae are not shown due to the uncertain position within Hedylopsacea and the different and special excretory system [59]). While Microhedylacea present a simple excretory system with a small, sac-like kidney (type I), hedylopsacean taxa evolved a complex excretory system with a large, internally divided kidney (type II): type IIa is characterised by a short nephroduct, type IIb by a long, looped nephroduct. The complex kidney already evolved in the ancestor of the Hedylopsacea. The mosaic-like distribution of habitat and excretory system types within Hedylopsacea implies an evolutionary scenario with multiple habitat shifts and adaptations. Abbreviations: **ao**, aorta; **h**, heart; **k**, kidney; **kn**, narrow lumen of kidney; **kw**, wide lumen of kidney; **nd**, nephroduct; **ndd**, dorsal branch of nephroduct; **ndv**, ventral branch of nephroduct; **pc**, pericardium. Not to scale.

To visualise patterns and reconstruct evolution in a more comprehensive context, habitats were plotted on a consensus tree (Fig. 12) combining all relevant acochlidian clades from morphology-based and molecular analyses. While the ancestral acochlidian [28] and all microhedylacean species are marine, the Hedylopsacea clade includes a mosaic of limnic, marine and brackish water associated taxa, implying several independent incidents of habitat shifts from marine to limnic and brackish water systems and/or vice versa. In contrast to previous assumptions [17], [18], the hedylopsacean ancestor could have been either still marine or already limnic.

In order to decide on a preferred scenario, we explored different characteristics and organ systems that are most closely linked to osmolarity changes. The first one is the body volume as a whole. Since all acochlidians, including all marine species and the basal limnic *Tantulum elegans* are small sized meiofaunal forms, there is no doubt that the large adult size of limnic, benthic Acochlidiidae is an adaptive apomorphy of this clade. The brackish water *Pseudunela espiritusanta* that is no more mesopsammic but living under stones either independently increased to an intermediate size or, alternatively, the common ancestor of *Pseudunela* plus Acochlidiidae already was large, with secondary reduction in mesopsammic *Pseudunela* species. Summing up, increasing body size alone may be advantageous but not strictly necessary for acochlidians invading freshwater or brackish water systems.

The second feature that is crucial for dealing with osmotic stress, especially in small species and juveniles, is the excretory system. Neusser & Schrödl [43] emphasised that the acochlidian excretory system varies considerably between marine and limnic species. The different types are illustrated in Fig. 12 and, based on our results, mapped on the consensus tree. All microhedylacean Acochlidia known in detail (e.g. *Microhedyle remanei*, *Pontohedyle milaschewitchii* (Kowalevsky, 1901) or *Asperspina murmanica*) have a quite simple excretory system of type I consisting of a small, sac-like kidney and a short nephroduct (Fig. 12) [14], [55], [60]. This simple type of sac-like kidney corresponds to almost all marine euthyneurans, including marine Panpulmonata, such as Siphonarioidea [61], the sacoglossan *Platyhedyle* [62], Amphiboloidea [63], and marine eupulmonates. In contrast, the acochlidian excretory system type II comprises a complex, internally divided kidney with a narrow and a wide lumen. All fully marine hedylopsacean species (such as the newly described *Pseudunela* species) have an excretory system of type II (Fig. 12), i.e. with a complex kidney, and with a short nephroduct (type IIa). *Hedylopsis ballantinei* Sommerfeldt & Schrödl, 2005 was described with a long, sac-like kidney and a nephropore opening into a mantle cavity [64], [65]. However, a brief re-examination of the original sections revealed this species to possess a complex, internally divided kidney (own unpubl. data). The most complex excretory system type IIb consists of a large, divided kidney as in type IIa, and additionally a long looped nephroduct with two branches. This type is present in all limnic acochlidian species, i.e. the small Caribbean limnic *Tantulum elegans* [66] and the large Indo-Pacific Acochlidiidae [44], in the brackish *Pseudunela espiritusanta* [43] and the at least temporary brackish *P. cornuta* [17]. Thus, the type of the excretory system in acochlidians is not strictly correlated with the habitat in acochlidian species: marine acochlidian species have either a type I or IIa excretory system with a simple or a complex kidney, respectivly.

Interestingly, all (marine) microhedylacean species have the simple, supposedly ancestral type I system. In contrast, all hedylopsacean species have the complex type II excretory system, even the marine species. We therefore conclude that the ancestral hedylopsacean species already had a complex kidney, which is an apomorphy of the clade. The presence of complex kidneys can be seen as a preadaptation to brackish water or limnic life, or much more likely, evolved as an adaptation to invading such habitats. Thus, considering evidence from excretory systems, we favour a scenario with hedylopsaceans originating in a freshwater, or at least freshwater influenced, habitat.

Considering the still poorly known and enigmatic Aitengidae [59] aberrant amphibious hedylopsacean offshoot (Fig. 11) would fit with and further extend the ecological tolerance and evolutionary plasticity observed within the hedylopsacean lineage.

Finally, the question arises if the complex type II kidney has already evolved in the – then supposedly brackish water or even limnic - ancestor of the Acochlidia. A recent multi-locus molecular study including six out of seven acochlidian families in a comprehensive euthyneuran taxon sampling [28] fundamentally changed our understanding of euthyneuran systematics. Surprisingly, this study confirms the Acochlidia in a well-supported (pan)pulmonate rather than opisthobranch relationship, as sister of basally still marine Eupulmonata. However, there is an alternative, though less likely topology suggesting that Acochlidia are the sister of – limnic – Hygrophila. In this scenario, a common ancestor could have been limnic as well, with a simple or complex kidney as both conditions occur apparently among different hygrophilan subgroups [61], [67], [68].

### Conclusions

Our study on mesopsammic Acochlidia testing the power of traditional taxonomy (i.e. examination of the external morphology and the radula) against results from in-depth micro-anatomical and molecular data clearly shows: 1) Traditional taxonomy fails to reveal the cryptic diversity within the genus *Pseudunela* in tropical sands, and thus is likely to generally underestimate biodiversity of meiofaunal invertebrates; 2) labour intensive and sophisticated 3D-modelling of micro-morphology is more suitable to delineate species, i.e. diagnosable units within *Pseudunela* are congruent with genetic lineages, and show relatively high genetic divergence; 3) only the combined evidence of microanatomical and molecular data enabled us to uncover and describe the full range of cryptic speciation in our material; low genetic distances of anatomically distinguishable genetic lineages of *P. viatoris* sp. nov. suggest there could be some gene flow between geographically distant populations, preventing us from establishing separate species; 4) patterns of distribution of *Pseudunela* species are discovered that cannot, however, be satisfyingly explained in the absence of sound biological knowledge on tiny meiofaunal species. We thus agree with Cook et al. [69] and advocate that taxonomy should integrate and consider all relevant types of data. Our exploration of the genus *Pseudunela* in older studies [17], [43] and herein also showed considerable ecological and structural diversity, i.e. of fully marine species, and those steadily or temporarily exposed to freshwater, having complex excretory systems. The combination of molecular phylogenetic and detailed micromorphological studies will shed further light on the origin of acochlidians, their much more frequent than expected habitat shifts, and their evolutionary adaptations to an extraordinarily wide range of completely different habitats.

## Supporting Information

Figure S1 **Interactive 3D-model of** ***Pseudunela viatoris*** **sp. nov. from Fiji.** To activate the 3D-model of *P. viatoris* sp. nov. for interactive manipulation click into figure. Rotate model by dragging with left mouse button pressed, shift model: same action+ctrl (or change default action for left mouse button), zoom: use mouse wheel. Select or deselect (or change transparency of) components in the model tree, switch between prefab views or change surface visualization (e.g. lightning, render mode, crop etc.). Interactive manipulation requires Adobe Reader 7 or higher.
(PDF)
