# Supplementary material for: Cryptic Species in Tropic Sands - Interactive 3D Anatomy, Molecular Phylogeny and Evolution of Meiofaunal Pseudunelidae (Gastropoda, Acochlidia)
Source: PLoS One. 2011 Aug 31;6(8):e23313. doi: 10.1371/journal.pone.0023313 (PMC3166138; doi:10.1371/journal.pone.0023313)
Supplement: Figure S1 — Interactive 3D-model of Pseudunela viatoris sp. nov. from Fiji. To activate the 3D-model of P. viatoris sp. nov. for interactive manipulation click into figure. Rotate model by dragging with left mouse button pressed, shift model: same action+ctrl (or change default action for left mouse button), zoom: use mouse wheel. Select or deselect (or change transparency of) components in the model tree, switch between prefab views or change surface visualization (e.g. lightning, render mode, crop etc.). Interactive manipulation requires Adobe Reader 7 or higher. (PDF) [file pone.0023313.s001.pdf]

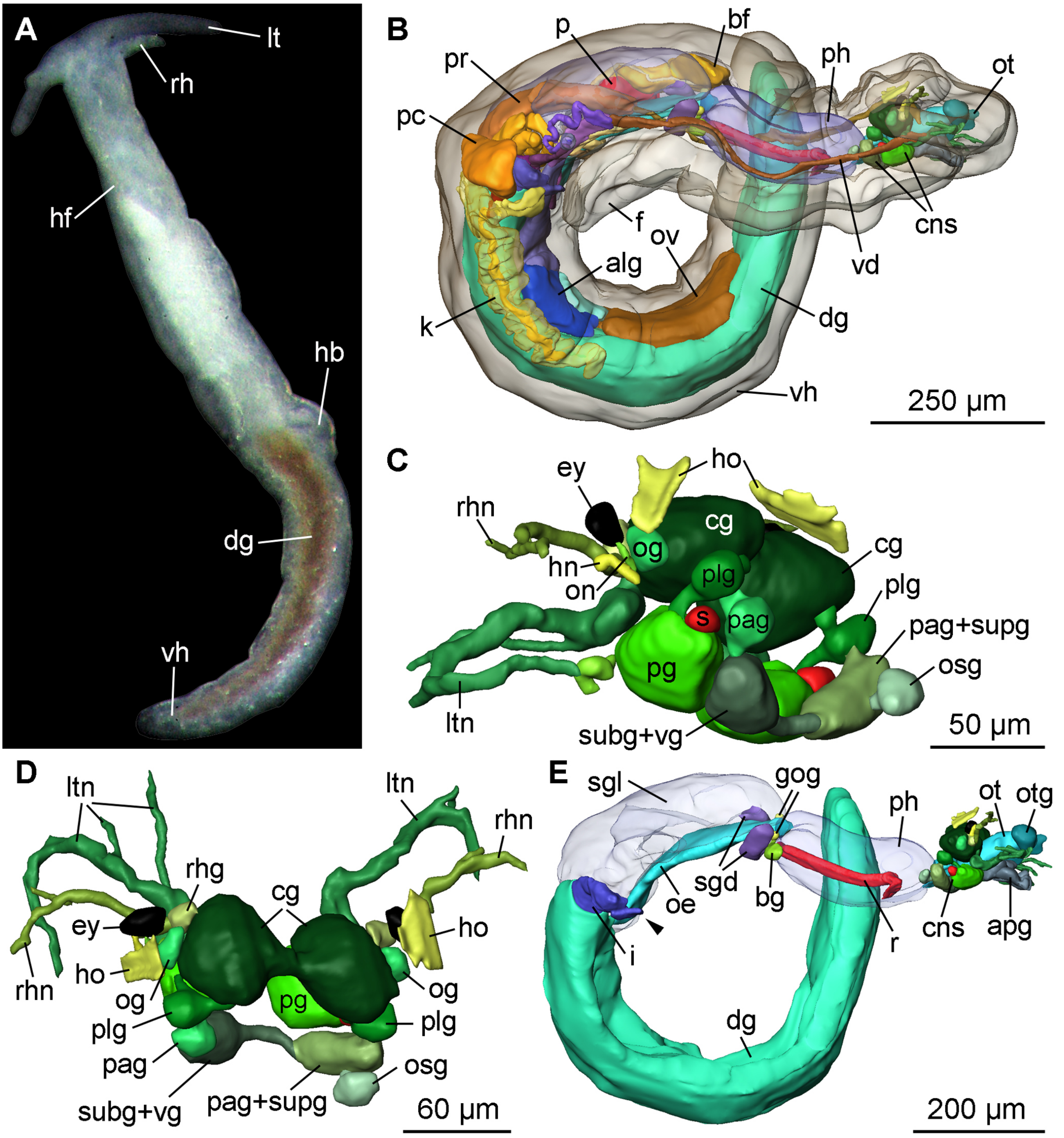

Figure 1. Photograph of a living specimen and 3D reconstruction of *P. viatoris* sp. nov. from Fiji.
A: external morphology of a living specimen (body size 3 mm), dorsal view. B: general anatomy, right view. C: CNS, left view. D: CNS, dorsal view. E: digestive system with CNS, right view. Abbreviations: **alg**, albumen gland; **apg**, anterior pedal gland; **bf**, basal finger; **bg**, buccal ganglion; **cg**, cerebral ganglion; **cns**, central nervous system; **dg**, digestive gland; **ey**, eye; **f**, foot; **gog**, gastro-oesophageal ganglion; **hb**, heart bulb; **hf**, head-foot complex; **hn**, Hancock`s nerve; **ho**, Hancock`s organ; **i**, intestine; **k**, kidney; **lt**, labial tentacle; **ltn**, labial tentacle nerve; **oe**, oesophagus; **og**, optic ganglion; **on**, optic nerve; **osg**, osphradial ganglion; **ot**, oral tube; **otg**, oral tube gland; **ov**, ovotestis; **p**, penis; **pag**, parietal ganglion; **pc**, pericardium; **pg**, pedal ganglion; **ph**, pharynx; **plg**, pleural ganglion; **pr**, prostate; **r**, radula; **rh**, rhinophore; **rhg**, rhinophoral ganglion; **rhn**, rhinophoral nerve; **s**, statocyst; **sgd**, salivary gland duct; **sgl**, salivary gland; **subg**, subintestinal ganglion; **supg**, supra-intestinal ganglion; **vd**, vas deferens; **vg**, visceral ganglion; **vh**, visceral hump; **arrowhead**, common opening of digestive and excretory systems. **The interactive 3D-model** of *P. viatoris* sp. nov. can be accessed by clicking onto Fig. 1 in the 3D PDF version of this article (Adobe Reader Version 7 or higher required). Rotate model by dragging with left mouse button pressed, shift model: same action + ctrl (or change default action for left mouse button), zoom: use mouse wheel. Select or deselect (or change transparency of) components in the model tree, switch between prefab views or change surface visualization (e.g. lightning, render mode, crop etc.).
